# Influence of Functional Additives with Potential Antimicrobial Properties on the Mechanical Performance and Accelerated Aging Resistance of (Meth)Acrylate-Based Composites

**DOI:** 10.3390/polym18182295

**Published:** 2026-09-19

**Authors:** Karolina Kiełczewska-Klim, Andrzej Puszka, Magdalena Jaszek, Beata Podkościelna

**Affiliations:** 1 Department of Polymer Chemistry, Institute of Chemical Sciences, Faculty of Chemistry, Maria Curie-Sklodowska University in Lublin, Gliniana 33, 20-614 Lublin, Poland; andrzej.puszka@mail.umcs.pl; 2 Department of Biochemistry and Biotechnology, Institute of Biological Sciences, Maria Curie-Sklodowska University in Lublin, Akademicka 19, 20-033 Lublin, Poland; magdalena.jaszek@mail.umcs.pl

**Keywords:** polymer composite materials, comonomers, special additives, mechanical properties, accelerated-aging test

## Abstract

The main aim of this study was to prepare and evaluate the physicochemical properties of new composite materials based on bisphenol A dimethacrylate modified with selected comonomers, namely 2-ethylhexyl acrylate (AEH), methyl methacrylate (MMA), 2-hydroxyethyl methacrylate (HEMA), and N-vinylpyrrolidone (NVP), and additives with potential antimicrobial properties, including nanosilver (Ag), copper(II) sulfate (CuSO_4_), benzethonium chloride (BEN), zinc oxide (ZnO), and zinc methacrylate (metZnO). The resulting composites were evaluated for their mechanical, structural, and ageing-related properties. The results demonstrated that the type of comonomer significantly influenced material performance. The lowest swelling coefficients were observed in composites containing AEH, whereas the highest values were observed in NVP-based materials. All investigated composites exhibited high hardness values. Accelerated ageing resulted in a reduction in hardness for all materials. Dynamic Mechanical Analysis (DMA) revealed that the highest glass transition temperatures were obtained for materials containing NVP and HEMA, particularly those modified with metZnO. Fourier Transform Infrared Spectroscopy (FTIR) confirmed that the chemical structure of the composites remained unchanged after accelerated ageing. Furthermore, colour changes were observed after ageing, with the most pronounced effects found for composites containing CuSO_4_ and BEN. The obtained results confirm that the appropriate selection of a comonomer and a modifying additive effectively enhances the beneficial properties of composite materials with special features.

## 1. Introduction

Polymer composites are widely investigated as materials in which selected properties can be tailored through controlled modification of the polymer matrix. The incorporation of additional components may influence the mechanical, thermal, optical, chemical, or biological characteristics of the resulting material. The extent and direction of these changes depend on the properties of both the matrix and the incorporated component, as well as on their concentration, distribution, and interactions within the composite structure [1,2,3]. Consequently, the incorporation of a functional component may affect several material properties simultaneously. A modification introduced to obtain a particular functionality may therefore also influence stiffness, hardness, thermal behaviour, moisture uptake, or resistance to environmental exposure. Understanding these relationships is essential for the rational design of polymer composites with defined performance [4,5].

Particularly interesting in this context are crosslinked methacrylate-based materials, which offer considerable flexibility in controlling the properties of the resulting polymer network through the selection of the monomer composition. The chemical structure of the monomer components, their relative proportions, and the conditions of polymerisation affect the formation of the crosslinked network and, consequently, the mobility of polymer-chain segments and the mechanical and thermomechanical behaviour of the material [6,7]. The use of reactive diluents is one of the approaches that can be employed to modify the composition and characteristics of such networks. Differences in molecular structure, polarity, and flexibility of the reactive diluent may affect the organisation of the polymer network and its response to external stimuli. These differences may become particularly important when the same functional additive is introduced into polymer matrices based on chemically different reactive diluents [8,9].

The modification of methacrylate-based polymers with functional components provides an opportunity to introduce additional properties while maintaining the basic characteristics of the polymer matrix [10]. Depending on their chemical structure and intended function, additives may influence the resistance of polymer materials to environmental factors, thermal behaviour, surface properties, or biological activity [11]. An important group of functional modifications involves the incorporation of antimicrobial components. Such modification is of interest for polymer materials that may be used in environments where surfaces are frequently touched or exposed to microorganisms. Functional polymer coatings may therefore be considered for applications involving worktops, cabinet elements, handles, or housings of laboratory and research equipment, where reducing microbial contamination may provide an additional benefit [12].

A number of chemically different compounds have been investigated for the preparation of antimicrobial polymer materials. Metallic and metal-oxide-based components, copper-containing compounds, and organic antimicrobial substances such as quaternary ammonium compounds represent distinct groups of modifiers that can provide antimicrobial activity through different mechanisms. Silver nanoparticles and zinc oxide have been widely studied as antimicrobial components of polymeric materials, while copper-containing compounds and quaternary ammonium derivatives provide alternative approaches to introducing antimicrobial functionality [13,14,15,16]. However, the incorporation of such components does not necessarily affect only the biological properties of the material. Changes in the mechanical and thermomechanical behaviour of the polymer matrix may also occur as a consequence of modifier incorporation. The magnitude and direction of these changes may depend on the chemical nature of the additive, its concentration and physical form, and its distribution within the polymer network [17,18,19].

The mechanical behaviour of modified polymer composites is particularly important because functionalisation may alter the organisation of the polymer network and, consequently, its resistance to deformation. Hardness provides information on the resistance of the material to local indentation, whereas dynamic mechanical analysis allows changes in stiffness and molecular relaxation to be evaluated over a range of temperatures [20,21]. Parameters such as storage modulus, loss modulus, glass transition temperature, and the width of the transition may provide complementary information on the influence of matrix composition and incorporated components on the structure and mobility of the polymer network. In addition, the long-term stability of functional composites should be considered because exposure to radiation, heat, and moisture may progressively alter their physical properties [22]. Changes in water uptake, mass, hardness, colour, or chemical structure may occur during environmental exposure and may differ substantially depending on the composition of the polymer matrix [23].

Previous studies on composite materials modified with functional additives possessing antimicrobial properties have typically focused on individual modifiers or specific polymer matrix formulations, evaluating selected mechanical, antimicrobial, or thermomechanical properties. However, systematic comparisons of structurally different modifiers incorporated into methacrylate networks containing different reactive diluents remain limited. In particular, the extent to which the chemical nature of the reactive diluent governs the effect of a given modifier on the mechanical and thermomechanical stability of the resulting polymer network during prolonged environmental exposure remains insufficiently understood. This issue is particularly important for functional polymer composites because the incorporation of an additive with antimicrobial properties may alter the structure and mechanical behavior of the polymer network in addition to providing the intended functional effect. Therefore, the performance of such materials cannot be assessed solely on the basis of their initial properties; the stability of the matrix–modifier system under prolonged environmental exposure must also be considered.

The aim of this study was therefore to investigate the effect of five functional additives with potential antimicrobial properties—nanosilver (Ag), copper(II) sulfate (CuSO_4_), benzethonium chloride (BEN), zinc oxide (ZnO), and zinc methacrylate (metZnO)—on the mechanical, structural, and thermomechanical properties of composite materials based on bisphenol A glycerolate dimethacrylate (BPA.DM) and four different reactive diluents: 2-ethylhexyl acrylate (AEH), methyl methacrylate (MMA), 2-hydroxyethyl methacrylate (HEMA), and N-vinyl-2-pyrrolidone (NVP). Particular attention was paid to determining whether the effects of the additives were consistent across different polymer matrices or depended on the chemical nature of the reactive diluent. The study combined Shore D hardness measurements, accelerated ageing, moisture-associated mass changes, ATR-FTIR spectroscopy, visual color assessment, and dynamic mechanical analysis (DMA) to evaluate the relationships between composite composition, network structure, mechanical properties, and ageing behavior.

The main contribution of this work is the direct comparison of five chemically different functional additives across four BPA.DM-based polymer matrices, allowing the matrix-dependent effects of the modifiers on mechanical performance and ageing behavior to be distinguished. The combined analysis of mechanical, structural, and thermomechanical parameters enables the effects of matrix composition and modifier type to be considered simultaneously rather than independently. This approach provides a more comprehensive understanding of how the matrix–modifier combination governs the performance and ageing behavior of BPA.DM-based functional composites.

## 2. Materials and Methods

### 2.1. Chemicals

Benzethonium chloride was obtained from Lonzagard^®^ (Basel, Switzerland). Zinc oxide, copper sulfate, silver nanoparticles (nanosilver), zinc methacrylate, methyl methacrylate (MMA), 2-ethylhexyl acrylate (AEH), 2-hydroxyethyl methacrylate (HEMA), N-vinyl-2-pyrrolidone (NVP), bisphenol A glycerolate dimethacrylate (BPA.DM), and 2,2-dimethoxy-2-phenylacetophenone (IQ) were purchased from Sigma-Aldrich (Taufkirchen, Germany).

### 2.2. Synthesis

According to the procedure described in the patent application (Patent No. 444108 [24]), a series of composite materials was prepared. The composite materials were prepared using bisphenol A glycerolate dimethacrylate (BPA.DM) as the base monomer and UV-induced polymerization. The BPA.DM was combined with one of the following four reactive diluents: 2-ethylhexyl acrylate (AEH), methyl methacrylate (MMA), N-vinyl-2-pyrrolidone (NVP), or 2-hydroxyethyl methacrylate (HEMA). The mass ratio of BPA.DM to the active diluent was 7:3. The resulting monomer mixtures were heated to 65 °C in a heating chamber to facilitate homogenization and degassing. Five functional additives with potential antimicrobial properties were used: nanosilver (Ag), copper(II) sulfate (CuSO_4_), benzethonium chloride (BEN), zinc oxide (ZnO), and zinc methacrylate (metZnO). Each additive was incorporated into the monomer mixture at concentrations of 1%, 5%, and 10% by weight relative to the total mass of the monomer mixture. For each polymer matrix, a corresponding composite without a functional additive was prepared as a control sample. The UV photoinitiator IQ was then added to the formulations. The amount of IQ was adjusted according to the modifier concentration: 1 wt.% for unmodified and 1 wt.% modifier composites, 2 wt.% for 5 wt.% modifier composites, and 3 wt.% for 10 wt.% modifier composites, relative to the monomer content. The formulations were thoroughly mixed and transferred into glass molds coated with an anti-stick agent and equipped with Teflon spacers. The molds were placed in a chamber with 160-watt mercury lamps (Eureka, Lublin, Poland) and exposed to UV radiation for 30 min. After UV irradiation, the molds were transferred to a heating chamber and maintained at 85 °C for four hours to complete the crosslinking process.

### 2.3. Methods

#### 2.3.1. ATR-FTIR Spectroscopy

The chemical structure of the composite materials before and after accelerated ageing was characterized using Fourier-transform infrared spectroscopy with attenuated total reflectance (ATR-FTIR). Measurements were performed using a Bruker TENSOR 27 FTIR spectrophotometer (Bruker Optics, Leipzig, Germany) equipped with an ATR accessory. Spectra were recorded in the range of 4000–600 cm^−1^. For each sample, 32 scans were collected at a spectral resolution of 4 cm^−1^. The spectra obtained before and after accelerated ageing were compared with respect to the positions and relative intensities of the characteristic absorption bands.

#### 2.3.2. Shore Hardness

The hardness of the composite materials was determined using the Shore D method with a Zwick 7206/04 hardness tester (ZwickRoell, Ulm, Germany). Ten measurements were performed for each material at 23 °C. Hardness values were recorded 15 s after indenter application in accordance with the relevant ISO standard [25].

#### 2.3.3. Accelerated Ageing

Accelerated ageing of the composite materials was carried out using a xenon arc weathering simulator (Atlas Xenotest^®^ Alpha+—Atlas Material Testing Technology GmbH, Linsengericht, Germany). The ageing procedure was performed under the following conditions: chamber temperature of 38 °C, irradiance of 60 W m^−2^, relative humidity of 50%, and a water-spray cycle consisting of 18 min of spraying followed by 102 min without spraying. The total exposure time was 1000 h and was divided into two consecutive ageing cycles of 500 h each. The specimens selected for accelerated ageing contained 10 wt.% of the respective functional additive relative to the total monomer content. This concentration corresponded to the highest modifier content investigated in the study and was therefore selected for subsequent ageing and thermomechanical characterization. Unmodified composites without functional additives were used as reference materials.

Before exposure, all specimens were weighed and photographed, and their chemical structure and hardness were evaluated by ATR-FTIR spectroscopy and Shore D measurements, respectively. After the first 500 h ageing cycle, the specimens were weighed and photographed again. They were then dried at 100 °C for 2 h to remove moisture absorbed during exposure. Following drying, the specimens were reweighed and analysed by ATR-FTIR spectroscopy. The mass loss upon drying (Δm, g) was calculated according to Equation (1):

Δm = *m_wet_* − *m_dry_*, (1)

where Δm is the mass loss upon drying (g), m_wet_ is the mass of the specimen immediately after ageing, and m_dry_ is the mass of the specimen after drying.

The specimens were subsequently subjected to a further 500 h of accelerated ageing. After completion of the second ageing cycle, the same weighing, drying, and ATR-FTIR analysis procedure was repeated. Shore D hardness measurements were also performed after 1000 h of exposure to evaluate changes in the mechanical properties associated with prolonged ageing.

#### 2.3.4. Dynamic Mechanical Analysis

Dynamic mechanical analysis (DMA) was performed using a DMA Q800 analyzer (TA Instruments, New Castle, DE, USA) equipped with a dual-cantilever clamp. DMA measurements were performed using the same 10 wt.% formulations selected for accelerated ageing. The measurements were conducted under ambient air conditions using a strain-controlled procedure with a displacement amplitude of 10 μm. Rectangular specimens with approximate dimensions of 2 ± 0.1 mm in thickness, 10 ± 0.2 mm in width, and 35 ± 0.1 mm in length were used for the measurements. Prior to the measurement, the specimens were equilibrated at −20 °C for 3 min. The temperature was subsequently increased from −20 to 185 °C at a constant heating rate of 3 °C/min. The temperature dependence of the storage modulus (E′), loss modulus (E″), and damping factor (tan δ) was recorded. The glass transition temperature (Tg) was determined from the maximum of the tan δ curve.

## 3. Results and Discussion

### 3.1. Hardness Determination

**Figure 1:**
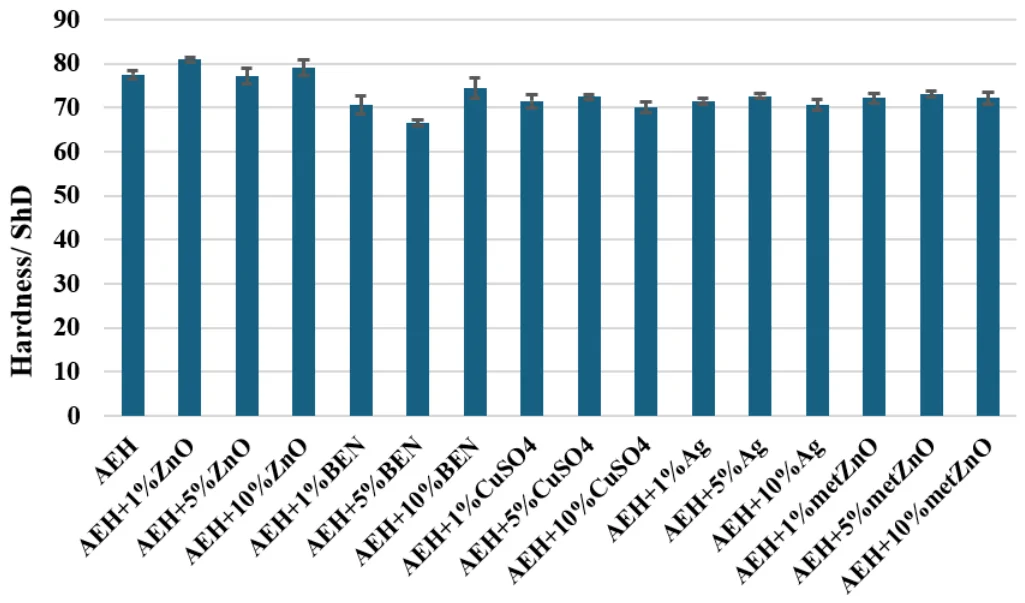
Shore D hardness of composites containing AEH as a reactive diluent and modified with selected functional additives. Values represent mean ± standard deviation (*n* = 10).

In the first stage of the study, the hardness of the obtained composite materials was determined. Measurements were performed in accordance with the relevant ISO standard. For each material, ten measurements were conducted, and the mean value together with the standard deviation was calculated. The results obtained for composites containing AEH are presented in Figure 1.

The Shore D hardness of the reference AEH-based material was 77.6 ± 1.0 ShD. The incorporation of 1 wt.% and 10 wt.% zinc oxide resulted in a slight increase in hardness, reaching 80.9 ± 0.6 ShD and 79.1 ± 1.8 ShD, respectively. In contrast, the addition of the remaining modifiers generally led to a reduction in hardness. The most pronounced decreases were observed for AEH+5%BEN (66.5 ± 0.7 ShD), AEH+10%CuSO_4_ (70.1 ± 1.3 ShD), AEH+1% BEN (70.6 ± 2.0 ShD), and AEH+10% Ag (70.6 ± 1.3 ShD).

No clear relationship was observed between either the concentration or the type of modifier and the hardness of the AEH-containing composite materials.

**Figure 2:**
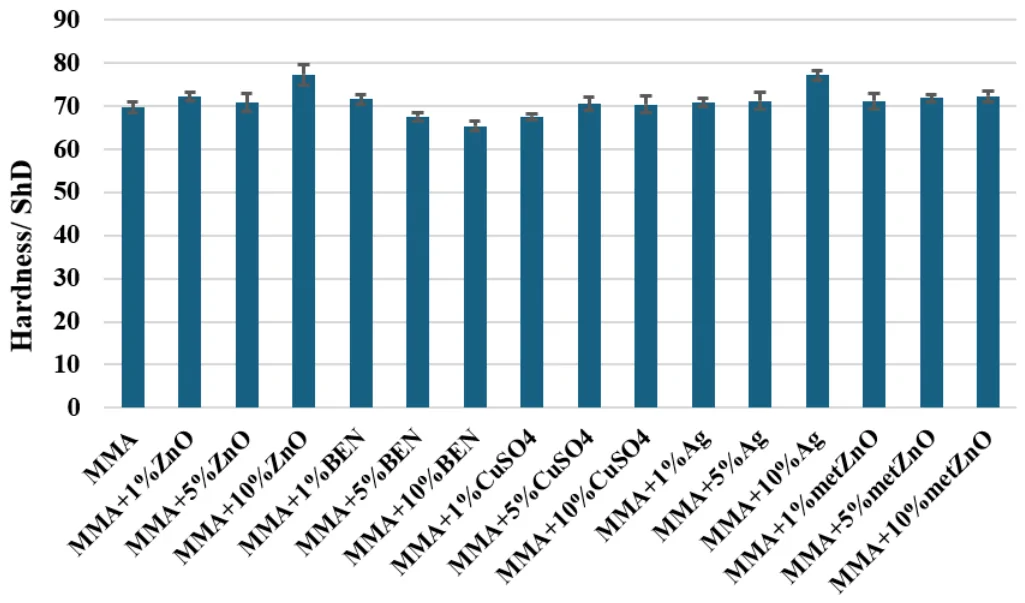
Shore D hardness of composites containing MMA as a reactive diluent and modified with selected functional additives. Values represent mean ± standard deviation (*n* = 10).

For the reference composite containing MMA, the Shore D hardness was 69.7 ± 1.2 ShD. The incorporation of 5 wt.% and 10 wt.% BEN, as well as 1 wt.% CuSO_4_, resulted in a slight decrease in hardness. The lowest hardness value was recorded for MMA+10%BEN (65.3 ± 1.2 ShD). In the case of the remaining modifiers, their incorporation into the composite material led to an increase in hardness. This effect was particularly pronounced for MMA+10%Ag (77.1 ± 1.0 ShD) and MMA+10%ZnO (77.3 ± 2.3 ShD). The obtained results are presented in Figure 2.

**Figure 3:**
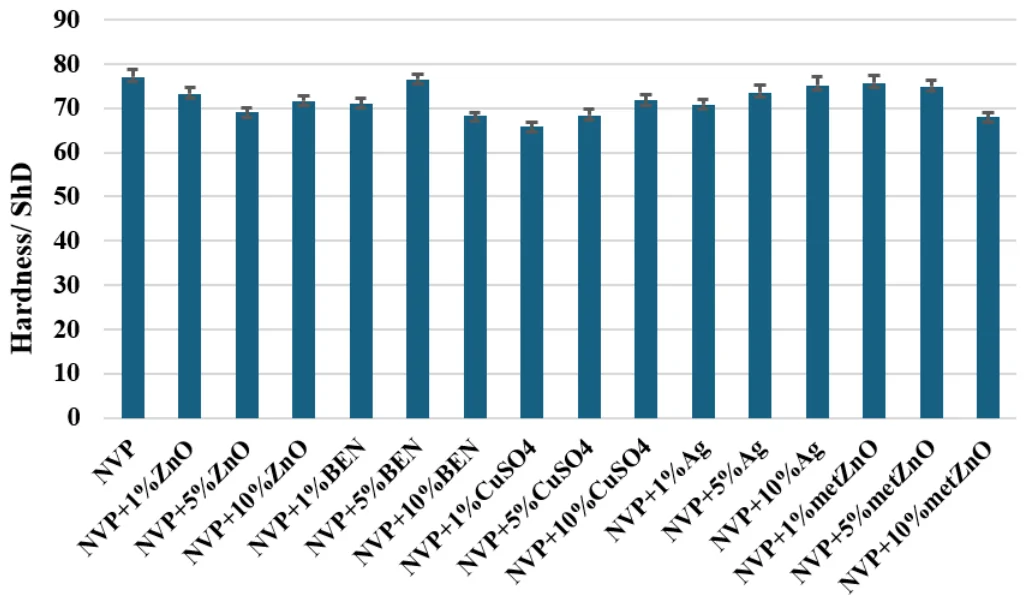
Shore D hardness of composites containing NVP as a reactive diluent and modified with selected functional additives. Values represent mean ± standard deviation (*n* = 10).

For the reference composite based on NVP, the Shore D hardness was 77.0 ± 1.9 ShD. All modified composites exhibited lower hardness values. The most pronounced reductions were observed for NVP+1%CuSO_4_ (65.8 ± 1.1 ShD) and NVP+10%metZnO (68.0 ± 1.1 ShD). Similar to the results obtained for the other materials, no clear relationship between the type or concentration of the modifier and the hardness of the composite was observed. The obtained results are presented in Figure 3.

**Figure 4:**
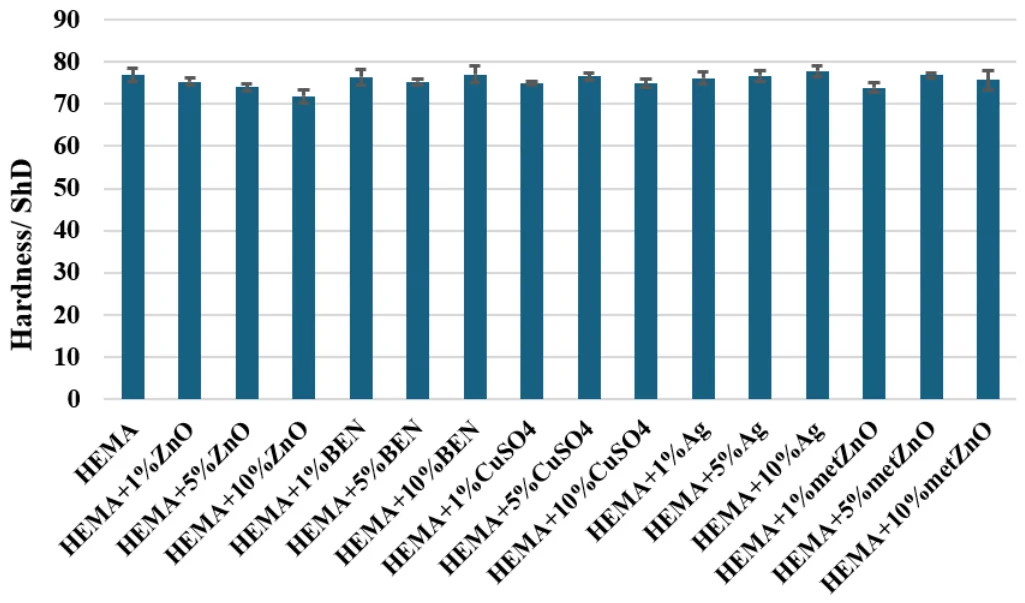
Shore D hardness of composites containing HEMA as a reactive diluent and modified with selected functional additives. Values represent mean ± standard deviation (*n* = 10).

The reference composite containing HEMA exhibited a Shore D hardness of 76.9 ± 1.7 ShD. For the materials HEMA+5%metZnO, HEMA+10%BEN, and HEMA+10%Ag, the hardness values were higher than those of the reference sample, reaching 76.9 ± 0.6 ShD, 77.0 ± 2.1 ShD, and 77.8 ± 1.3 ShD, respectively. The remaining composites exhibited lower hardness values than the reference material. The lowest hardness value recorded among the tested samples was 71.8 ± 1.5 ShD for HEMA + 10%ZnO (Figure 4).

The observed differences in Shore D hardness indicate that the effect of the functional additives was strongly dependent on both the polymer matrix and the type and concentration of the modifier. The largest decreases in hardness were observed for the NVP+1%CuSO_4_ and AEH+5%BEN composites, with reductions of 11.2 and 11.1 ShD, respectively, compared with the corresponding unmodified materials. In the MMA-based system, the decrease was considerably smaller, with the largest reduction of 4.4 ShD observed for MMA+10%BEN, whereas in the HEMA matrix the largest decrease was 5.1 ShD for HEMA+10%ZnO. These differences indicate that the chemical structure of the polymer matrix strongly influenced its response to modifier incorporation. The decrease in hardness may result from changes in the local organization of the polymer network and increased mobility of polymer segments, which reduce resistance to indentation. It may also occur when incorporation of the modifier disturbs the continuity of the polymer network or reduces the efficiency of load transfer within the composite. The particularly pronounced decrease observed for AEH+5%BEN may be related to the effect of benzethonium chloride on the local intermolecular interactions within the AEH-based network, which may reduce its resistance to indentation. The strong decrease observed for NVP+1%CuSO_4_ may indicate that the copper sulfate additive affected the local organization of the NVP-based network, resulting in a greater reduction in hardness than that observed in the other matrices. The particularly pronounced effects of BEN in the AEH matrix and CuSO_4_ in the NVP matrix therefore suggest that the response of the polymer network depended on the specific combination of matrix and modifier. In contrast, the increase in hardness observed for selected MMA- and AEH-based composites, particularly those containing Ag or ZnO, indicates that some modifiers may additionally contribute to indentation resistance, depending on their incorporation and distribution within the polymer matrix. Thus, the effect of the additives cannot be considered as universally reinforcing, but rather as a matrix-dependent change in the mechanical response of the composite.

The hardness results confirmed that the hardness of the composite materials was influenced by both the type of modifier and the composition of the polymer matrix. However, despite the observed variations in Shore D hardness values, no clear relationship was identified between modifier content and composite hardness. These results imply that the mechanical response of the materials under investigation is determined by various structural factors, such as the nature of the polymer matrix, the modifier’s distribution within the composite structure, and the interactions at the polymer–filler interface. Similar observations have been reported in previous studies, demonstrating that the influence of modifiers on the mechanical performance of polymer composites depends on their concentration, dispersion, and interactions with the polymer network. For example, Hammani et al. showed that incorporating ZnO nanoparticles altered the morphology, and therefore the mechanical properties, of PMMA/PEG blends. Conversely, Su et al. demonstrated that the mechanical behavior of silver nanoparticle-reinforced polyethylene was strongly affected by nanoparticle dispersion and interfacial interactions between the filler and the polymer matrix. These results are consistent with those of the present study, indicating that the hardness of the investigated composites is determined by the combined effects of the polymer matrix composition, the distribution of the filler, and the interactions between the polymer and the modifier rather than by the presence or concentration of a specific antimicrobial modifier alone [26,27].

For composites modified with ZnO, increasing the additive content did not consistently result in a proportional increase in hardness. Similar observations have previously been reported for polymer composites containing inorganic modifiers. A beneficial reinforcing effect was observed only within a specific concentration range of the additives. At higher concentrations, the reinforcing efficiency may be limited by poorer dispersion of the modifier and the formation of local structural heterogeneities within the material. Harun-Ur-Rashid et al. emphasised that achieving a homogeneous distribution of nanoparticles is essential for improving the mechanical performance of polymer nanocomposites containing silver nanoparticles, whereas agglomeration of the particles may diminish their reinforcing effect [19,26]. The higher hardness observed for the composite containing 1 wt.% ZnO may be related to the reinforcing effect of the rigid inorganic additive and its possible restriction of local segmental mobility within the polymer network. However, the further increase in ZnO concentration to 10 wt.% did not result in a proportional increase in hardness. This non-linear response may be associated with changes in the local network structure and/or the distribution of the modifier within the matrix at higher additive concentrations. At higher ZnO contents, the efficiency of reinforcement may therefore be reduced by the formation of local heterogeneities or less efficient stress transfer within the composite.

For composites modified with nanosilver, an increase in hardness was observed primarily in materials based on MMA and HEMA. Previous studies have indicated that well-dispersed silver nanoparticles can improve the mechanical performance of polymeric materials. However, the magnitude of this effect depends strongly on the nature of the polymer matrix, highlighting the importance of interactions between the modifier and the polymer network [28].

The effect of copper(II) sulfate was less consistent and was found to depend on the type of polymer matrix. In contrast, the incorporation of benzethonium chloride generally resulted in a reduction in hardness compared with the corresponding unmodified materials. Similar observations have been reported for polymer systems containing quaternary ammonium compounds (QACs), whose presence may adversely affect the structural organization of the material and consequently lead to deterioration of selected mechanical properties. Chladek et al. reported that incorporating quaternary ammonium-containing nanoparticles influences the physical and mechanical properties of experimental dental composites by altering polymer–filler interactions. Similarly, Chrószcz and Barszczewska-Rybarek showed that the impact of quaternary ammonium derivatives on composite properties hinges on their concentration and distribution within the polymer matrix [28,29]. While benzethonium chloride differs structurally from the quaternary ammonium compounds examined in these studies, it still belongs to the QAC family. Consequently, the observed reduction in hardness may also be the result of disturbances in polymer network organization and altered intermolecular interactions following the incorporation of modifiers.

### 3.2. Accelerated Aging Test

**Figure 5:**
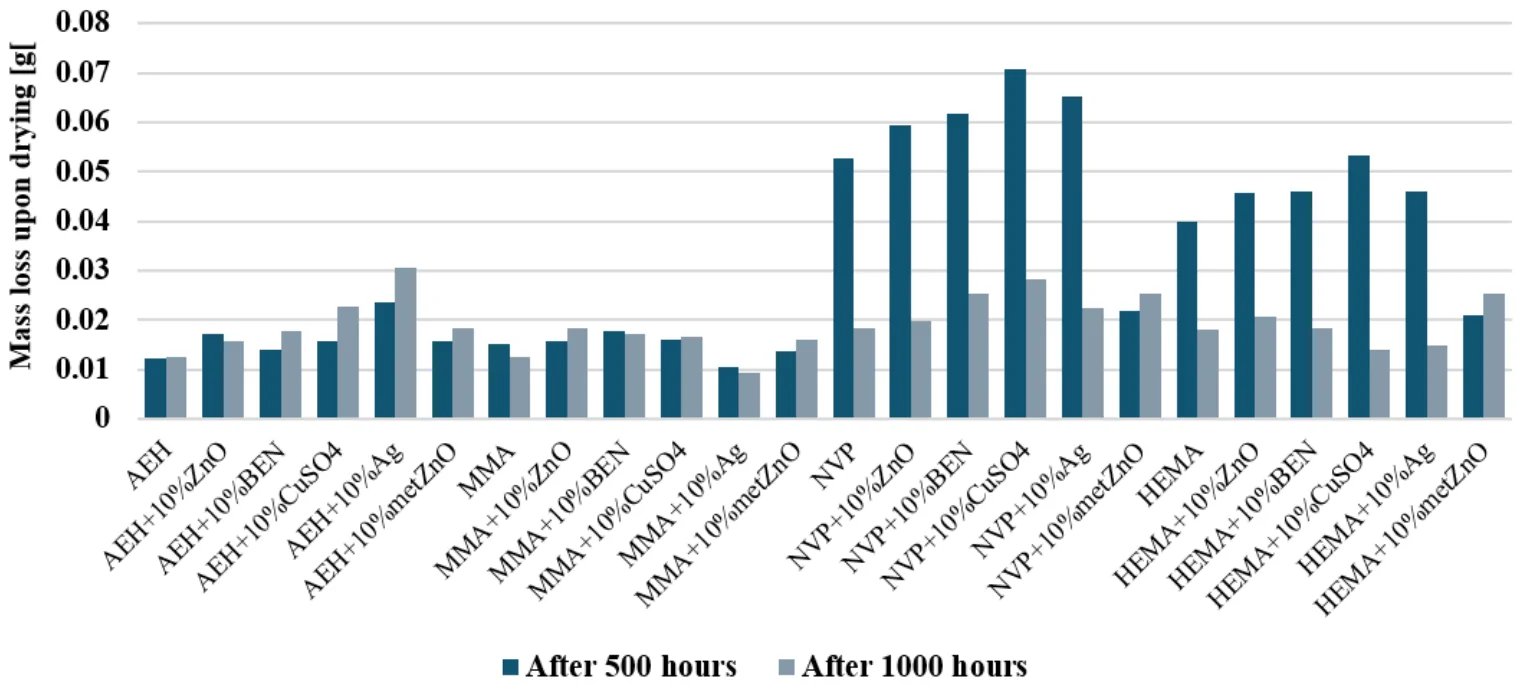
Mass loss upon drying (Δm, g) of the investigated composite materials after 500 and 1000 h of accelerated ageing.

The water loss upon drying (Δ*m*), representing the amount of moisture absorbed by the polymer network during accelerated ageing and subsequently removed through thermal drying (100 °C, 2 h), is presented in Figure 5. The overall sorption capacity demonstrated a strong dependence on the chemical nature of the polymer matrix (AEH, MMA, NVP, and HEMA) and the total exposure duration (500 h and 1000 h).

For composite materials in which AEH and MMA were used as reactive diluents, low values of Δm were maintained throughout the study for all composite samples. For samples containing AEH, a minor increase in mean mass loss was recorded: from 0.0164 g after 500 h to 0.0196 g after 1000 h. Within this group, the composite modified with nanosilver (AEH+10% Ag) exhibited the highest water absorption, reaching 0.0237 g after 500 h and 0.0307 g after 1000 h. For materials containing MMA, very similar average Δm values were observed: 0.0148 g after 500 h and 0.0149 g after 1000 h. The MMA+10% Ag sample exhibited the lowest total moisture absorption, reaching 0.0093 g after 1000 h.

Composites containing NVP and HEMA as reactive diluents demonstrated a significant increase in water absorption after the initial 500-h cycle, reaching average values of 0.0553 g and 0.0420 g, respectively. Introducing copper(II) sulfate produced the highest moisture retention capacity: NVP+10%CuSO_4_ = 0.0706 g and HEMA+10%CuSO_4_ = 0.0532 g. Extending the exposure time to 1000 h caused a sharp, more than twofold drop in Δm values for nearly all NVP and HEMA formulations. This lowered the average values to 0.0232 g for NVP and 0.0185 g for HEMA. The only exception to this decline was observed in samples containing zinc methacrylate (metZnO), which exhibited stable Δm values across both time intervals.

Analysis of the mass loss upon drying indicates that the behavior of the investigated materials in an aqueous environment was influenced primarily by the type of comonomer used. Among the analyzed polymer systems, the lowest Δ*m* values after 500 h of exposure were observed for materials containing AEH, whereas the highest values were recorded for NVP-based composites. These findings suggest that the ability of the materials to absorb the surrounding medium was governed to a greater extent by the chemical nature of the polymer matrix than by the type of modifier incorporated into the composite.

The low susceptibility to water absorption observed for AEH-containing materials may be attributed to the presence of more hydrophobic structural moieties and their lower affinity toward water. In contrast, the higher Δ*m* values determined for NVP-based composites are consistent with the properties of polymer systems containing pyrrolidone groups, which exhibit high polarity and a strong ability to interact with water molecules [30,31]. Similar relationships have previously been reported for methacrylate-based materials modified with hydrophilic monomers, where an increased concentration of polar groups resulted in enhanced water sorption [32].

No clear relationship was observed between either the type or concentration of the modifier and the mass loss upon drying value. It is noteworthy that for AEH-containing materials, the Δ*m* values generally increased with prolonged contact time with the ageing medium. In contrast, the opposite trend was observed for composites containing NVP, HEMA, and MMA. The decrease in Δ*m* values after extended exposure may indicate that the initial absorption of the medium was partially compensated for by simultaneous leaching of low-molecular-weight components. Similar phenomena have previously been reported for crosslinked polymeric materials exposed to aqueous environments, where an initial increase in mass associated with liquid sorption was followed by stabilization or even a decrease in the measured parameters as a consequence of the extraction of species not permanently incorporated into the polymer network [33,34,35]. The drying step applied after each 500 h ageing interval should also be considered when interpreting the obtained results. The specimens were dried at 100 °C for 2 h primarily to remove moisture absorbed during the ageing process and to allow determination of the dry mass. Because the same drying procedure was applied to all investigated composites, the obtained mass changes can be compared between the different formulations. However, a possible influence of thermal conditioning at 100 °C on the polymer network cannot be completely excluded. Since the effect of the drying treatment was not investigated separately in the present study, its individual contribution to the observed changes cannot be quantitatively determined. Therefore, the drying step was considered primarily as a conditioning procedure for moisture removal, and its possible influence should be considered when interpreting the results.

In the next stage of the study, changes in the hardness of the composite materials after 1000 h of accelerated ageing were evaluated. The obtained results are presented in Table 1. A decrease in hardness was observed for all composite materials following the ageing process.

Among the AEH-based composites, the largest decrease in hardness was observed for the material containing 10% nanosilver. The hardness decreased from 79.1 ± 1.8 ShD before ageing to 71.9 ± 1.4 ShD after ageing. The smallest change was recorded for AEH+10%BEN, for which the hardness decreased from 72.2 ± 1.3 ShD to 71.7 ± 2.1 ShD. For composites containing MMA as the comonomer, the greatest reduction in hardness was observed for MMA+10%metZnO, whereas the smallest change was recorded for MMA+10%BEN. The hardness values for MMA+10%metZnO decreased from 77.1 ± 1.0 ShD to 69.2 ± 0.6 ShD, and for MMA+10%BEN decreased from 72.3 ± 1.3 ShD to 71.9 ± 1.4 ShD. Among the NVP-based composites, the smallest change in hardness was observed for NVP+10%metZnO, while the largest decrease was recorded for the unmodified NVP material. The hardness values changed from 75.2 ± 1.9 ShD to 74.8 ± 1.4 ShD and from 77.0 ± 1.9 ShD to 75.5 ± 4.1 ShD, respectively. For the HEMA-based composites, the smallest difference in hardness was determined for HEMA+10%CuSO_4_, for which the hardness decreased from 77.0 ± 2.1 ShD to 76.5 ± 1.9 ShD. The largest decrease was observed for HEMA+10%metZnO, where the hardness declined from 77.8 ± 1.3 ShD to 70.0 ± 1.6 ShD.

A characteristic phenomenon observed in crosslinked polymeric materials subjected to prolonged environmental exposure is a reduction in hardness. This effect may be associated with medium sorption, plasticization of the polymer matrix, and structural changes occurring within the polymer network during the ageing process [33,36]. Ferracane demonstrated that water absorbed by crosslinked methacrylate networks acts as a plasticizer. This increases polymer chain mobility, which consequently reduces the material’s mechanical properties. Similarly, Sideridou et al. found that the extent of water absorption depends heavily on the resin matrix’s chemical composition, with more hydrophilic systems being more susceptible to property deterioration. Although an overall decreasing trend in hardness was observed for all investigated materials, the magnitude of the changes was strongly dependent on both the type of comonomer and the modifier used [34].

For AEH-based materials, the greatest reduction in hardness was observed for the composite modified with nanosilver, whereas the smallest decrease was recorded for the material containing benzethonium chloride. In the case of MMA-based composites, the largest change was observed for materials modified with metZnO, while the smallest decrease occurred for composites containing benzethonium chloride. These findings indicate that additives that initially improve the mechanical properties of a material do not necessarily enhance its resistance to long-term ageing conditions. A similar relationship has been described for polymer composites undergoing hydrothermal aging. In this case, the initial mechanical performance does not directly predict long-term durability because aging-induced degradation is governed by water diffusion, polymer relaxation, and filler–matrix interactions. Qi et al. and Aceti et al. emphasized that the long-term behavior of composite materials depends on both their initial mechanical properties and the stability of the polymer network and polymer–filler interface during environmental exposure. These observations are consistent with the present findings [37,38].

The highest hardness stability during ageing was observed for composites containing NVP. Despite exhibiting relatively high water-associated mass changes after ageing, these materials showed the least reduction in hardness. This suggests that water retention alone is not the main factor that determines mechanical stability. Rather, resistance to hardness loss appears to depend on the polymer network’s ability to maintain its structural integrity during prolonged environmental exposure. The obtained results suggest that, despite their relatively high susceptibility to swelling, these materials maintained a high resistance to hardness loss during prolonged environmental exposure. In contrast, for HEMA-based composites, no clear relationship was observed between the type of modifier employed and the changes in hardness after ageing, indicating that the influence of the incorporated antimicrobial agents was secondary to that of the polymer matrix itself.

### 3.3. ATR-FTIR Analyses

**Figure 6:**
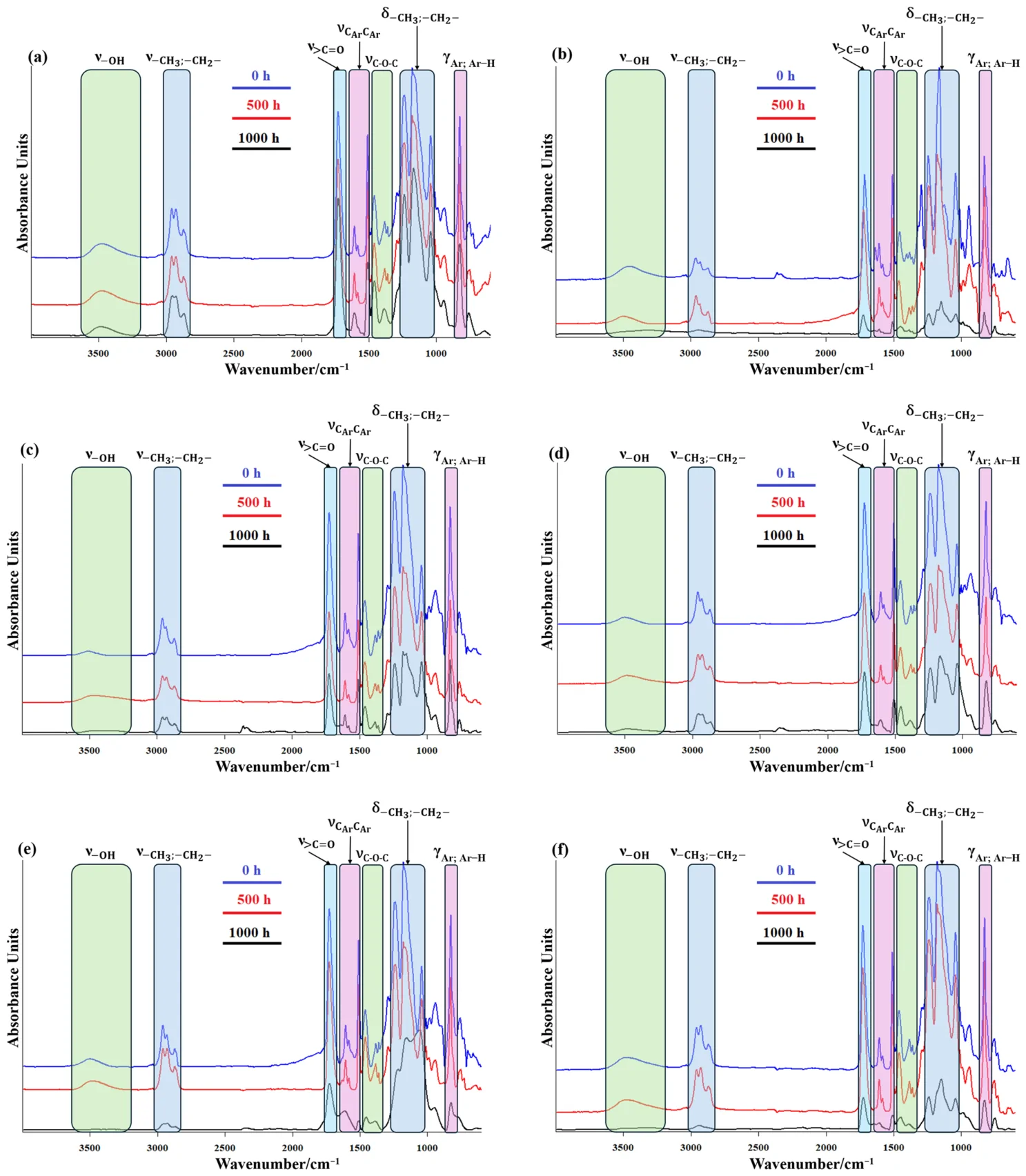
FTIR spectra of composite materials with AEH as an active diluent recorded before ageing and after 500 and 1000 h of accelerated ageing: (**a**) unmodified composite (control), (**b**) composite containing ZnO, (**c**) composite containing benzethonium chloride (BEN), (**d**) composite containing CuSO_4_, (**e**) composite containing nanosilver (Ag), and (**f**) composite containing zinc methacrylate (metZnO).

The next stage involved comparing structural changes in the materials before ageing and after 500 and 1000 h of accelerated ageing. The spectra are presented in Figure 6 and Figures S1–S3 (Supplementary Materials). Before ageing, the FTIR spectra of the composite samples exhibited bands attributed to out-of-plane aromatic ring vibrations (γCArH) at 830 cm^−1^. The bands observed at 1040, 1160, and 1250 cm^−1^ were assigned to C–O–C stretching vibrations. Bands corresponding to deformation vibrations of methyl and methylene groups were detected at 1390, 1416, and 1460 cm^−1^. Aromatic ring stretching vibrations were observed at 1510, 1615, and 1630 cm^−1^. The absorption band at 1730 cm^−1^ was assigned to carbonyl group (C=O) stretching vibrations. The bands located at 2930 and 2960 cm^−1^ corresponded to the stretching vibrations of –CH_2_– and –CH_3_ groups. The presence of hydroxyl groups was confirmed by a broad absorption band centred at approximately 3450 cm^−1^.

Analysis of the FTIR spectra recorded before ageing and after 500 and 1000 h of exposure demonstrated that the characteristic spectral profile of the crosslinked methacrylate matrix was generally preserved for most of the investigated composites. No new intense absorption bands indicative of extensive chemical degradation were observed. The detected changes were primarily associated with variations in the intensity of bands assigned to νOH, νC=O, and νC–O–C vibrations. These results may suggest that the aging process primarily affected the local environment of the functional groups and promoted sorption in the aging environment. The FTIR spectra obtained for the material after 1000 h of aging showed distinct changes in the intensity of these bands, suggesting significant changes in the chemical structures of the studied materials. Celina [39] emphasized that the early stages of polymer aging are often characterized by subtle spectral changes associated with oxidation, moisture absorption, and physical aging before extensive chemical degradation is detectable by FTIR spectroscopy. Similarly, Campanale et al. [40] demonstrated that FTIR spectroscopy is a sensitive tool for monitoring modifications induced by aging in polymeric materials. Changes in the intensity of the bands associated with hydroxyl groups (above 3300 cm^−1^) and carbonyl groups (1800–1600 cm^−1^) reflect changes in intermolecular interactions and exposure to environmental factors, and also indicate degradation, primarily hydrolysis of the ester bonds in the materials and their photodegradation. Therefore, the FTIR results obtained in this study suggest that the accelerated aging conditions used induced chemical changes in the tested methacrylate composites, as evidenced, among other things, by changes in the physical properties of the samples after aging (including changes in color).

**Figure 7:**
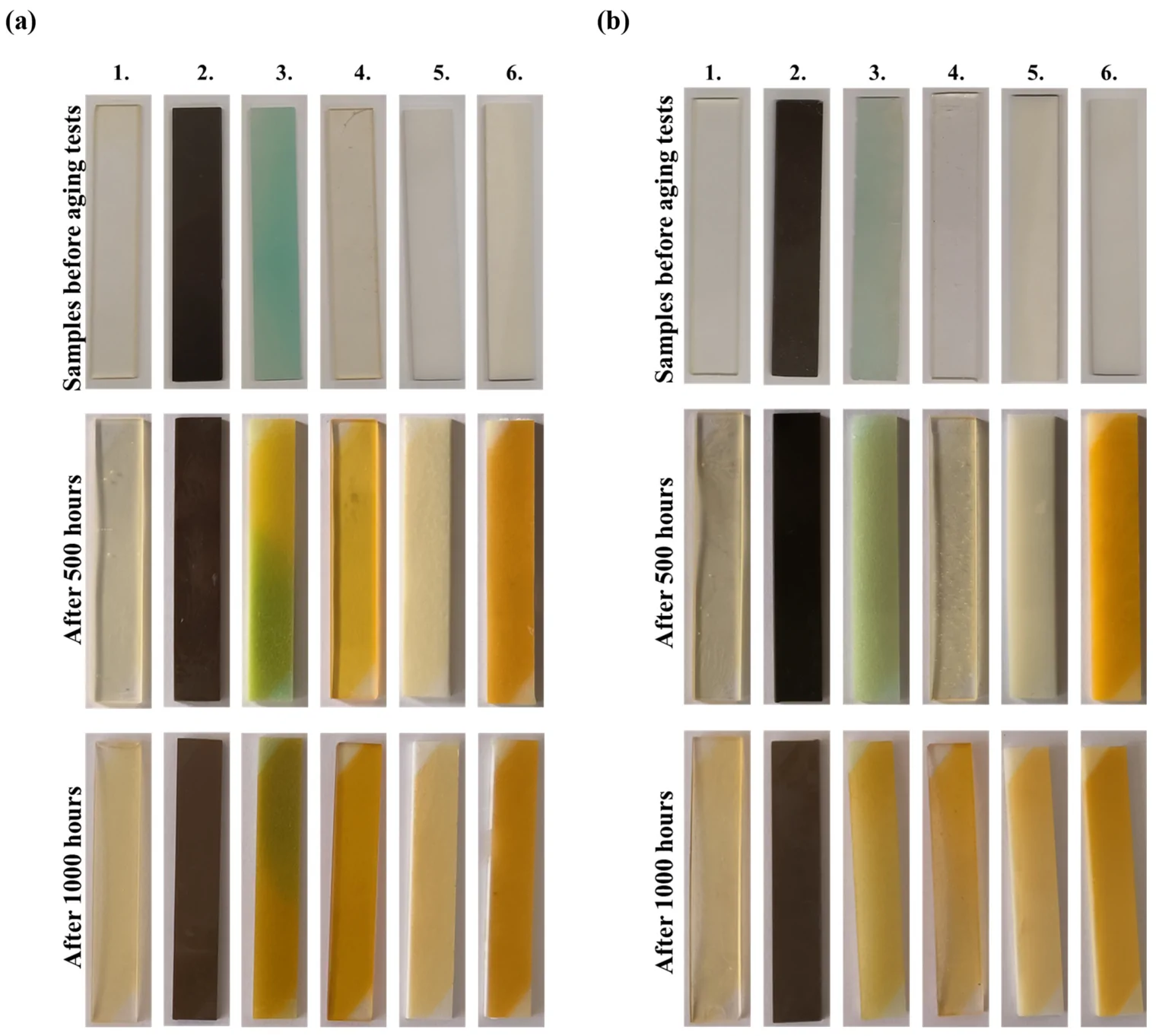
Color changes in HEMA- and NVP-based composite materials before ageing and after 500 and 1000 h of accelerated ageing. (**a**) HEMA-based composites; (**b**) NVP-based composites. Sample designations: (1) unmodified composite (control), (2) Ag, (3) CuSO_4_, (4) BEN, (5) ZnO, and (6) metZnO.

A visual assessment of colour changes occurring during the ageing process was also performed (Figure 7 and Figure S4—Supplementary Materials).

All investigated composites exhibited colour changes; however, the extent of discoloration depended on both the type of comonomer and the modifier used. Changes in the color of the samples during aging were most pronounced after the first irradiation stage, i.e., after 500 h. Further irradiation in the chamber did not result in any further noticeable changes in the colors of the resulting composites. The highest colour stability was observed for materials containing nanosilver. Although these samples exhibited a dark colour before ageing due to the presence of silver nanoparticles, no substantial visual changes were detected after 500 and 1000 h of exposure. For composites containing CuSO_4_ and BEN, the most pronounced colour changes were observed in materials based on MMA and AEH, whereas significantly smaller differences were found for HEMA- and NVP-based systems. A similar trend was observed for the unmodified reference materials. In the case of composites containing metZnO and ZnO, the initially milky white samples gradually changed colour to yellow and subsequently to yellow–orange with increasing ageing time. Comparison of the investigated systems indicated that composites containing NVP and HEMA exhibited superior colour stability. Nevertheless, irrespective of the matrix composition, progressive yellowing was observed with prolonged ageing. Such behaviour is typical of polymeric materials exposed to elevated temperature, humidity, and radiation for extended periods and is commonly associated with oxidation processes and the formation of degradation products. These processes may lead to the formation of chromophoric groups, including conjugated carbonyl-containing structures that absorb visible light and cause yellowing. Celina emphasized that discoloration is often one of the earliest visible signs of polymer aging and can occur without extensive chemical degradation detectable by spectroscopic techniques. Similarly, Ci et al. [41] demonstrated that prolonged UV exposure caused progressive yellowing of epoxy-based composites due to photo-oxidative degradation. This was accompanied by the gradual deterioration of the polymer structure and changes in its physicochemical properties. Therefore, the color changes observed in the present study suggest that accelerated aging induced progressive oxidative processes within the investigated methacrylate composites. However, the FTIR results indicate that these changes were predominantly limited to the early stages of aging and did not involve extensive degradation of the polymer network [39,41]. Based on the results obtained, it can also be concluded that the presence of fillers catalyzed changes in the color of the resulting composites.

### 3.4. Dynamic Mechanical Analysis

**Figure 8:**
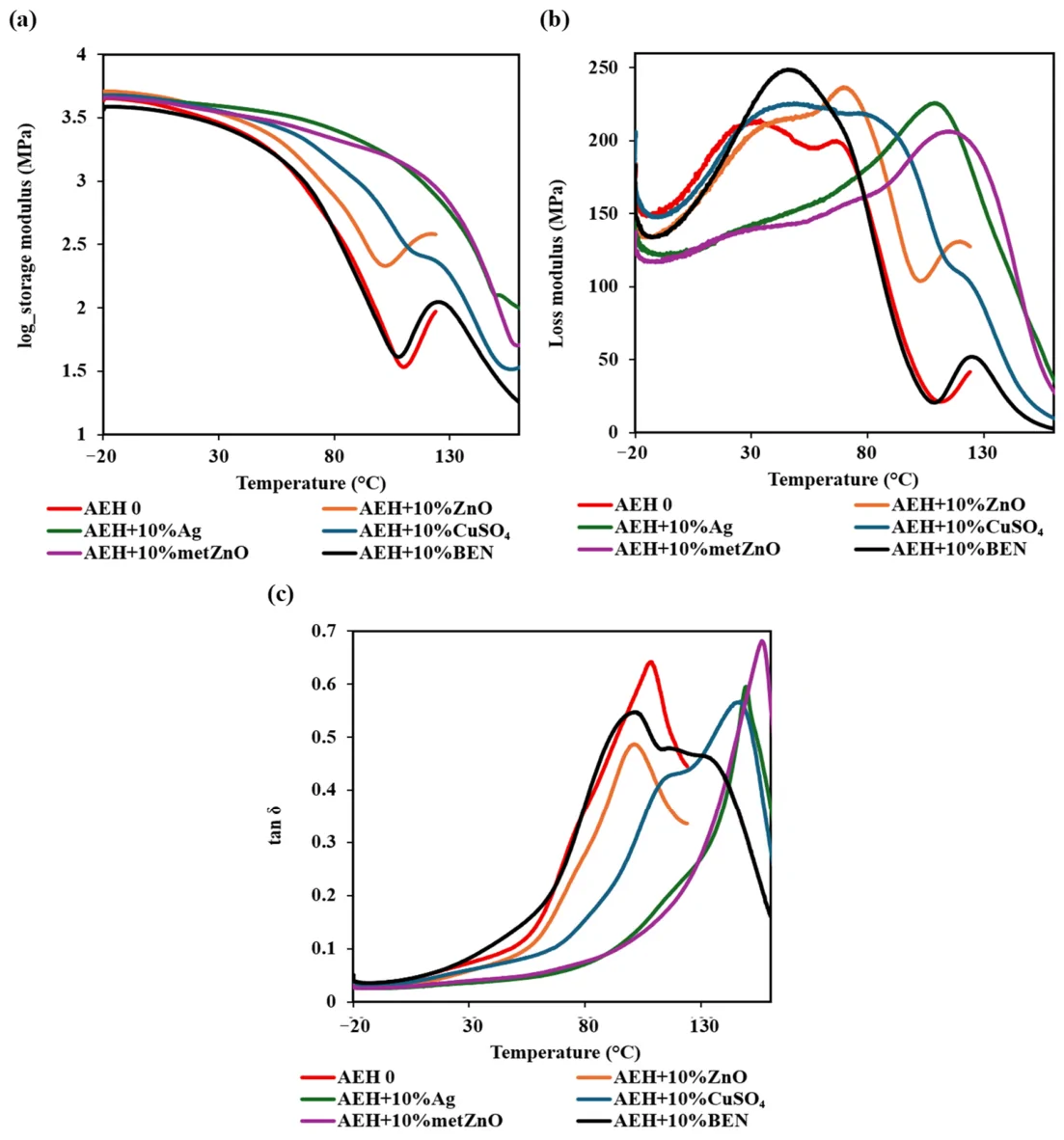
Dynamic mechanical analysis (DMA) curves of AEH-based composite materials: (**a**) storage modulus (E′), (**b**) loss modulus (E″), and (**c**) tangent delta (tan δ).

The parameters obtained from DMA analysis are summarized in Table 2, Figure 8, and Figures S5–S7 (Supplementary Materials). Material homogeneity was evaluated based on the full width at half maximum (FWHM) of the tanδ peak. Lower FWHM values indicate a more homogeneous structure, whereas increasing FWHM values reflect greater heterogeneity of the polymer network [42,43].

All of the materials that were investigated exhibited a characteristic decrease in storage modulus as the temperature increased, which corresponded to the glass transition of the polymer network. For some of the composites, a slight increase in E′ was observed between 110 and 130 °C. This behavior may be associated with post-curing and further development of the polymer network during heating. An increase in storage modulus in this temperature range may also be related to changes in the mobility of polymer-chain segments occurring during heating. The storage modulus at 20 °C (E′_20_) depended strongly on the composition of the polymer matrix and the type of incorporated antimicrobial modifier. The most pronounced increase in E′_20_ was observed for NVP-based composites. The value increased from 2305.42 MPa for the unmodified material to 4801.80 MPa after metZnO was incorporated. Considerable improvements were also observed for composites containing BEN (3654.34 MPa) and Ag (3434.19 MPa). The increase in E′_20_ observed after incorporation of these modifiers indicates an increase in the elastic response of the composites, which may be associated with reduced segmental mobility of the polymer chains and/or more effective stress transfer within the modified polymer network. In filled polymer systems, the magnitude of this effect may depend on the rigidity of the incorporated phase, its distribution within the matrix, and the interactions between the modifier and the polymer network. MMA-based composites generally exhibited higher storage modulus values following modification. The greatest reinforcing effect was recorded for Ag (4104.98 MPa) and CuSO_4_ (3905.03 MPa) compared to the reference material (3324.55 MPa). The increase in E′_20_ observed for these composites may therefore reflect a greater restriction of segmental motion and an increased contribution of the incorporated phase to the mechanical response of the matrix. In the AEH series, the most significant increase was observed with CuSO_4_ (2905.90 MPa), while ZnO slightly reduced the storage modulus compared to the unmodified composite (1959.40 vs. 2029.74 MPa). The different responses to ZnO and CuSO_4_ indicate that the presence of a functional additive does not necessarily produce a reinforcing effect. A decrease in E′_20_ may result when incorporation of the additive modifies the local organization of the polymer network or reduces the efficiency of stress transfer through the composite. In contrast, HEMA-based composites generally exhibited lower E′_20_ values after modification. While the storage modulus remained relatively high for Ag-containing materials (3913.02 MPa) and metZnO-containing materials (3718.83 MPa), none of the modified HEMA composites exceeded the reference sample’s value (4038.44 MPa). This contrasting behavior demonstrates that the mechanical effect of a given modifier depends on the polymer matrix in which it is incorporated. Differences in matrix structure and segmental mobility may determine whether the incorporated additive acts primarily as a reinforcing phase or instead contributes to local disruption of the network structure. Overall, these findings suggest that the efficiency with which the modifiers reinforce the polymer matrix is primarily governed by the composition of the matrix and the compatibility between the modifier and the matrix, rather than by the modifiers themselves.

For materials containing AEH as a reactive diluent, FWHM values ranged from 32.00 to 50.67 °C. The most homogeneous structure was observed for the unmodified reference material, whereas the addition of ZnO resulted in a considerable broadening of the tanδ peak. Among MMA-based composites, the widest variation in FWHM values was observed (25.16–54.18 °C). The highest structural homogeneity was found for the metZnO-modified material, while the lowest was recorded for the composite containing CuSO_4_. In HEMA-based materials, the lowest FWHM value was obtained for the Ag-modified composite, whereas the highest value was observed for the material containing metZnO. Similarly, in NVP-based systems, the narrowest tanδ peak was recorded for BEN-modified composites, while the broadest peak was observed for the metZnO-containing material. Bandzierz et al. [44] demonstrated that the width of the tanδ peak is closely related to the structural homogeneity of crosslinked polymer networks. Narrower peaks indicate a more uniform network structure, while broader peaks reflect increased heterogeneity resulting from a wider distribution of relaxation times. Similarly, Hearon et al. found that changes in crosslink density and network architecture significantly impact the breadth of the glass transition, confirming that the full width at half maximum (FWHM) is a useful indicator of polymer network homogeneity [44,45]. The broadening of the tanδ peak can therefore be associated with a greater diversity of local environments in the polymer network, in which polymer-chain segments experience different degrees of restriction and consequently relax over a wider temperature range. Such heterogeneity may arise from differences in local network structure and from the distribution of the incorporated modifier within the matrix. These results indicate that an increase in glass transition temperature was not necessarily accompanied by improved structural homogeneity, suggesting the simultaneous restriction of segmental mobility and an increase in network heterogeneity.

The temperature corresponding to the maximum of the loss modulus (E″max) was lower than the temperature determined from the tanδ maximum for all investigated materials, which is characteristic of crosslinked polymer systems. Mahna et al. [46] reported similar relationships for ZnO-reinforced dental composites, demonstrating that the E″ maximum precedes the tanδ maximum because these parameters describe different aspects of molecular relaxation during the glass transition. Menard also emphasized that E″ reflects the maximum rate of viscous energy dissipation, whereas the tanδ peak represents the balance between the elastic and viscous components of the viscoelastic response, and therefore occurs at slightly higher temperatures in crosslinked polymers [46,47]. Changes in the E″ maximum therefore indicate changes in the temperature at which the energy dissipation associated with molecular relaxation is most pronounced. An increase in the E″ maximum may reflect a greater contribution of viscous relaxation processes to the overall response of the composite. For AEH-containing composites, E″max values varied only slightly, indicating a limited influence of the additives on relaxation processes. In MMA- and HEMA-based materials, the highest E″max values were observed for composites containing nanosilver, whereas in NVP-based systems the highest value was recorded for the CuSO_4_-modified composite. The different responses of the modifiers in the individual matrices suggest that the magnitude of energy dissipation was determined not only by the incorporated additive but also by the molecular mobility and network structure of the polymer matrix. These observations indicate that the influence of the investigated antimicrobial modifiers on molecular relaxation processes was strongly dependent on the polymer matrix composition.

The glass transition temperature (Tg) determined from the tanδ maximum was strongly dependent on the type of comonomer used. The lowest Tg values were obtained for AEH-based materials (96.70–120.71 °C), indicating the highest mobility of polymer chain segments. Higher Tg values were observed for MMA-based composites, while a further increase was recorded for HEMA- and NVP-containing materials. Among all investigated systems, the highest glass transition temperature was determined for NVP+10%metZnO (188.38 °C). Regardless of the comonomer employed, the incorporation of metZnO resulted in some of the highest Tg values. The marked differences in Tg between the polymer matrices indicate that the chemical structure of the reactive diluent strongly affected the thermal activation of segmental relaxation. The particularly high Tg observed for NVP+10%metZnO may be related to the effect of metZnO on the local structure and mobility of the NVP-based polymer network. Overall, the DMA results suggest that the type of comonomer exerted a greater influence on the thermomechanical properties of the composites than the presence of the investigated functional additives.

## 4. Conclusions

In this study, BPA.DM-based composite materials containing AEH, MMA, HEMA, or NVP as reactive diluents and 1, 5, or 10 wt.% of selected functional additives with potential antimicrobial properties were investigated in terms of their mechanical, structural, thermomechanical, and ageing-related properties.

The results demonstrated that the chemical nature of the reactive diluent was an important factor determining the behaviour of the investigated composites. The effect of the functional additives on hardness was strongly matrix-dependent, and no general relationship between modifier concentration and hardness was observed. The largest reductions in initial hardness were observed for NVP+1%CuSO_4_ and AEH+5%BEN, whereas selected Ag- and ZnO-containing MMA and AEH composites exhibited increased hardness. These results indicate that the mechanical effect of a functional modifier depends on the specific polymer matrix and cannot be considered independently of its composition.

The ageing experiments showed a pronounced influence of the polymer matrix on moisture-associated mass changes. AEH-based composites exhibited the lowest mass changes, whereas NVP-based materials showed the highest values after 500 h of exposure. Despite their higher moisture-associated mass changes, NVP-based composites exhibited comparatively small hardness losses during prolonged ageing, indicating that moisture uptake alone did not determine the mechanical stability of the materials.

ATR-FTIR analysis showed that the characteristic spectral profile of the methacrylate matrix was generally preserved after accelerated ageing, although changes in selected absorption bands were observed. These results indicate that ageing affected the chemical environment of selected functional groups, but no evidence of extensive chemical degradation was observed under the applied conditions. Visual assessment additionally demonstrated that colour changes occurred during ageing, with the extent of discoloration depending on both the polymer matrix and the incorporated modifier.

DMA measurements further confirmed the strong influence of matrix composition on the thermomechanical behaviour of the composites. The NVP+10%metZnO material exhibited the highest E′_20_ and Tg values among the investigated systems, whereas the effect of the same modifier was less pronounced or reversed in other matrices. The FWHM values also demonstrated that the modifiers affected the homogeneity of the relaxation processes in a matrix-dependent manner. Overall, the results show that the mechanical and thermomechanical response of the investigated composites is governed by the combined effects of the reactive diluent and functional modifier rather than by the presence or concentration of the modifier alone.

## 5. Patent Applications

Młynarczyk, K., Podkościelna, B., Osińska-Jaroszuk, M., and Jaszek, M. (2023) [24] Method of obtaining a polymeric composite with antimicrobial activity and a polymeric composite obtained by this method. P. 444108, issued 2023.

## Figures and Tables

**Table 1:** Shore D hardness of the investigated composite materials before and after 1000 h of accelerated ageing (mean ± standard deviation, *n* = 10).

Composite	Hardness Before Aging Test [ShD]	Hardness After Aging Test [ShD]	ΔH [ShD]
AEH	77.6 ± 1.0	72.3 ± 1.8	−5.3
AEH+10%ZnO	70.1 ± 1.3	68.9 ± 2.3	−1.2
AEH+10%BEN	72.2 ± 1.3	71.7 ± 2.1	−0.5
AEH+10%CuSO_4_	74.4 ± 2.4	73.4 ± 2.5	−1.0
AEH+10%Ag	79.1 ± 1.8	71.9 ± 1.4	−7.2
AEH+10%metZnO	70.6 ± 1.3	68.4 ± 2.3	−2.2
MMA	69.7 ± 1.2	68.0 ± 2.2	−1.7
MMA+10%ZnO	70.3 ± 1.9	68.4 ± 1.0	−1.9
MMA+10%BEN	72.3 ± 1.3	71.9 ± 1.4	−0.4
MMA+10%CuSO_4_	65.3 ± 1.2	63.9 ± 2.2	−1.4
MMA+10%Ag	77.3 ± 2.3	75.0 ± 1.9	−2.3
MMA+10%metZnO	77.1 ± 1.0	69.2 ± 0.6	−7.9
NVP	77.0 ± 1.9	75.5 ± 4.1	−1.5
NVP+10%ZnO	71.8 ± 1.1	70.5 ± 2.2	−1.3
NVP+10%BEN	68.0 ± 1.1	66.7 ± 1.3	−1.3
NVP+10%CuSO_4_	68.2 ± 0.8	65.9 ± 1.9	−2.3
NVP+10%Ag	71.7 ± 1.1	70.7 ± 3.1	−1.0
NVP+10%metZnO	75.2 ± 1.9	74.8 ± 1.4	−0.4
HEMA	76.9 ± 1.7	74.6 ± 1.7	−2.3
HEMA+10%ZnO	74.9 ± 1.1	70.9 ± 2.6	−4.0
HEMA+10%BEN	75.7 ± 2.2	73.8 ± 3.0	−1.9
HEMA+10%CuSO_4_	77.0 ± 2.1	76.5 ± 1.9	−0.5
HEMA+10%Ag	71.8 ± 1.5	70.8 ± 2.4	−1.0
HEMA+10%metZnO	77.8 ± 1.3	70.0 ± 1.6	−7.8

**Table 2:** Selected dynamic mechanical analysis (DMA) parameters of the investigated composite materials.

Composite	E′_20_ [MPa]	E″_max_ [°C]	E″_max_ [MPa]	tanδ_max_ [°C]	tanδ_max_	FWHM [°C]
AEH *	2029.74	103.11	31.54	102.89	0.413	32.00
AEH+10%ZnO *	1989.64	55.81	139.14	102.84	0.356	50.67
AEH+10%BEN	2477.67	74.83	155.31	108.94	0.513	35.04
AEH+10%CuSO_4_	2905.90	105.50	140.91	105.64	0.325	41.89
		114.37	166.96			
AEH+10%Ag	2109.00	76.60	158.10	96.70	0.280	34.05
		112.74	168.20			
AEH+10%metZnO	2840.62	80.57	131.00	120.71	0.430	32.16
MMA *	3324.55	66.54	200.04	107.07	0.631	33.65
MMA+10%ZnO *	3967.99	71.22	236.37	100.22	154.22	27.01
		119.89	130.82			
MMA+10%BEN	3110.75	45.50	248.64	101.88	0.548	31.52
		124.65	52.11			
MMA+10%CuSO_4_	3905.03	51.00	225.12	145.53	0.563	54.18
MMA+10%Ag	4104.98	109.74	225.78	149.43	0.568	26.35
MMA+10%metZnO	3775.79	117.13	206.23	156.27	0.665	25.16
NVP	2305.42	64.99	124.10	99.29	0.339	34.51
		121.42	71.86			
NVP+10%ZnO	2518.02	67.56	146.70	146.71	0.264	31.93
NVP+10%BEN	3654,34	42.27	197.51	129.88	0.404	30.47
		74.80	192.49			
NVP+10%CuSO_4_	2809.88	84.41	151.80	163.44	0.385	41.55
NVP+10%Ag	3434.19	116.01	168.22	148.65	0.340	32.98
NVP+10%metZnO	4801.80	132.97	232.62	188.38	0.295	47.96
HEMA *	4038.44	70.87	240.34	104.47	0.635	38.18
HEMA+10%ZnO *	3466.29	48.01	308.40	67.40	0.289	30.15
HEMA+10%BEN	3587.15	98.17	259.67	139.80	0.841	31.65
HEMA+10%CuSO_4_	3197.85	84.46	220.24	128.51	0.706	36.98
HEMA+10%Ag	3913.02	103.16	263.42	149.60	0.847	25.44
HEMA+10%metZnO	3718.83	122.25	231.78	165.51	0.523	46.32

Note: * indicates composite materials that underwent mechanical failure before reaching 150 °C.

## Data Availability

The raw data supporting the conclusions of this article will be made available by the authors on request.

## Supplementary Materials

The following supporting information can be downloaded at: https://www.mdpi.com/article/10.3390/polym18182295/s1, Figure S1. FTIR spectra of composite materials with HEMA as an active diluent recorded before ageing and after 500 and 1000 h of accelerated ageing: (a) unmodified composite (control), (b) composite containing ZnO, (c) composite containing benzethonium chloride (BEN), (d) composite containing CuSO_4_, (e) composite containing nanosilver (Ag), and (f) composite containing zinc methacrylate (metZnO); Figure S2. FTIR spectra of composite materials with NVP as an active diluent recorded before ageing and after 500 and 1000 h of accelerated ageing: (a) unmodified composite (control), (b) composite containing ZnO, (c) composite containing benzethonium chloride (BEN), (d) composite containing CuSO_4_, (e) composite containing nanosilver (Ag), and (f) composite containing zinc methacrylate (metZnO); Figure S3. FTIR spectra of composite materials with MMA as an active diluent recorded before ageing and after 500 and 1000 h of accelerated ageing: (a) unmodified composite (control), (b) composite containing ZnO, (c) composite containing benzethonium chloride (BEN), (d) composite containing CuSO_4_, (e) composite containing nanosilver (Ag), and (f) composite containing zinc methacrylate (metZnO); Figure S4. Color changes of AEH- and MMA-based composite materials before ageing and after 500 and 1000 h of accelerated ageing. (a) AEH-based composites; (b) MMA-based composites. Sample designations: (1) unmodified composite (control), (2) Ag, (3) CuSO_4_, (4) BEN, (5) ZnO, and (6) metZnO; Figure S5. Dynamic mechanical analysis (DMA) curves of MMA-based composite materials: (a) storage modulus (E′), (b) loss modulus (E″), and (c) tangent delta (tan δ); Figure S6. Dynamic mechanical analysis (DMA) curves of NVP-based composite materials: (a) storage modulus (E′), (b) loss modulus (E″), and (c) tangent delta (tan δ); Figure S7. Dynamic mechanical analysis (DMA) curves of HEMA-based composite materials: (a) storage modulus (E′), (b) loss modulus (E″), and (c) tangent delta (tan δ).

## Abbreviations

The following abbreviations are used in this manuscript:

DMA	Dynamic mechanical analysis
AEH	composite containing 2-ethylhexyl acrylate as a reactive diluent (without modifier)
AEH+10%ZnO	composite containing 2-ethylhexyl acrylate as a reactive diluent and zinc oxide
AEH+10%metZnO	composite containing 2-ethylhexyl acrylate as a reactive diluent and zinc methacrylate
AEH+10%Ag	composite containing 2-ethylhexyl acrylate as a reactive diluent and nanosilver
AEH+10%CuSO_4_	composite containing 2-ethylhexyl acrylate as a reactive diluent and copper(II) sulphate
AEH+10%BEN	composite containing 2-ethylhexyl acrylate as a reactive diluent and benzethonium chloride
MMA	composite containing methyl methacrylate as a reactive diluent (without modifier)
MMA+10%ZnO	composite containing methyl methacrylate as a reactive diluent and zinc oxide
MMA+10%metZnO	composite containing methyl methacrylate as a reactive diluent and zinc methacrylate
MMA+10%Ag	composite containing methyl methacrylate as a reactive diluent and nanosilver
MMA+10%CuSO_4_	composite containing methyl methacrylate as a reactive diluent and copper(II) sulphate
MMA+10%BEN	composite containing methyl methacrylate as a reactive diluent and benzethonium chloride
HEMA	composite containing 2-hydroxyethyl methacrylate as a reactive diluent (without modifier)
HEMA+10%ZnO	composite containing 2-hydroxyethyl methacrylate as a reactive diluent and zinc oxide
HEMA+10%metZnO	composite containing 2-hydroxyethyl methacrylate as a reactive diluent and zinc methacrylate
HEMA+10%Ag	composite containing 2-hydroxyethyl methacrylate as a reactive diluent and nanosilver
HEMA+10%CuSO_4_	composite containing 2-hydroxyethyl methacrylate as a reactive diluent and copper(II) sulphate
HEMA+10%BEN	composite containing 2-hydroxyethyl methacrylate as a reactive diluent and benzethonium chloride
NVP	composite containing N-vinylpyrrolidone as a reactive diluent (without modifier)
NVP+10%ZnO	composite containing N-vinylpyrrolidone as a reactive diluent and zinc oxide
NVP+10%metZnO	composite containing N-vinylpyrrolidone as a reactive diluent and zinc methacrylate
NVP+10%Ag	composite containing N-vinylpyrrolidone as a reactive diluent and nanosilver
NVP+10%CuSO_4_	composite containing N-vinylpyrrolidone as a reactive diluent and copper(II) sulphate
NVP+10%BEN	composite containing N-vinylpyrrolidone as a reactive diluent and benzethonium chloride
