# Supplementary material for: Influence of Functional Additives with Potential Antimicrobial Properties on the Mechanical Performance and Accelerated Aging Resistance of (Meth)Acrylate-Based Composites
Source: Polymers (Basel). 2026 Sep 19;18(18):2295. doi: 10.3390/polym18182295 (PMC13611347; doi:10.3390/polym18182295)
Supplement: Supplementary file 1 [file polymers-18-02295-s001.zip › polymers-4527278-supplementary.pdf]

polymers

MDPI

Article

Influence of Functional Additives with Potential Antimicrobial Properties on the Mechanical Performance and Accelerated Aging Resistance of (Meth)Acrylate-Based Composites

Karolina Kiełczewska-Klim [1,*], Andrzej Puszka [1], Magdalena Jaszek [2] and Beata Podkościelna [1,*]

[1] Department of Polymer Chemistry, Institute of Chemical Sciences, Faculty of Chemistry, Maria Curie-Sklodowska University in Lublin, Gliniana 33, 20-614 Lublin, Poland; andrzej.puszka@mail.umcs.pl
[2] Department of Biochemistry and Biotechnology, Institute of Biological Sciences, Maria Curie-Sklodowska University in Lublin, Akademicka 19, 20-033 Lublin, Poland; magdalena.jaszek@mail.umcs.pl
* Correspondence: karolina.mlynarczyk@mail.umcs.pl (K.K.-K.); beata.podkoscielna@mail.umcs.pl (B.P.)

Keywords: polymer composite materials; comonomers; special additives, mechanical properties; accelerated-aging test.

SUPPLEMENTARY MATERIALS

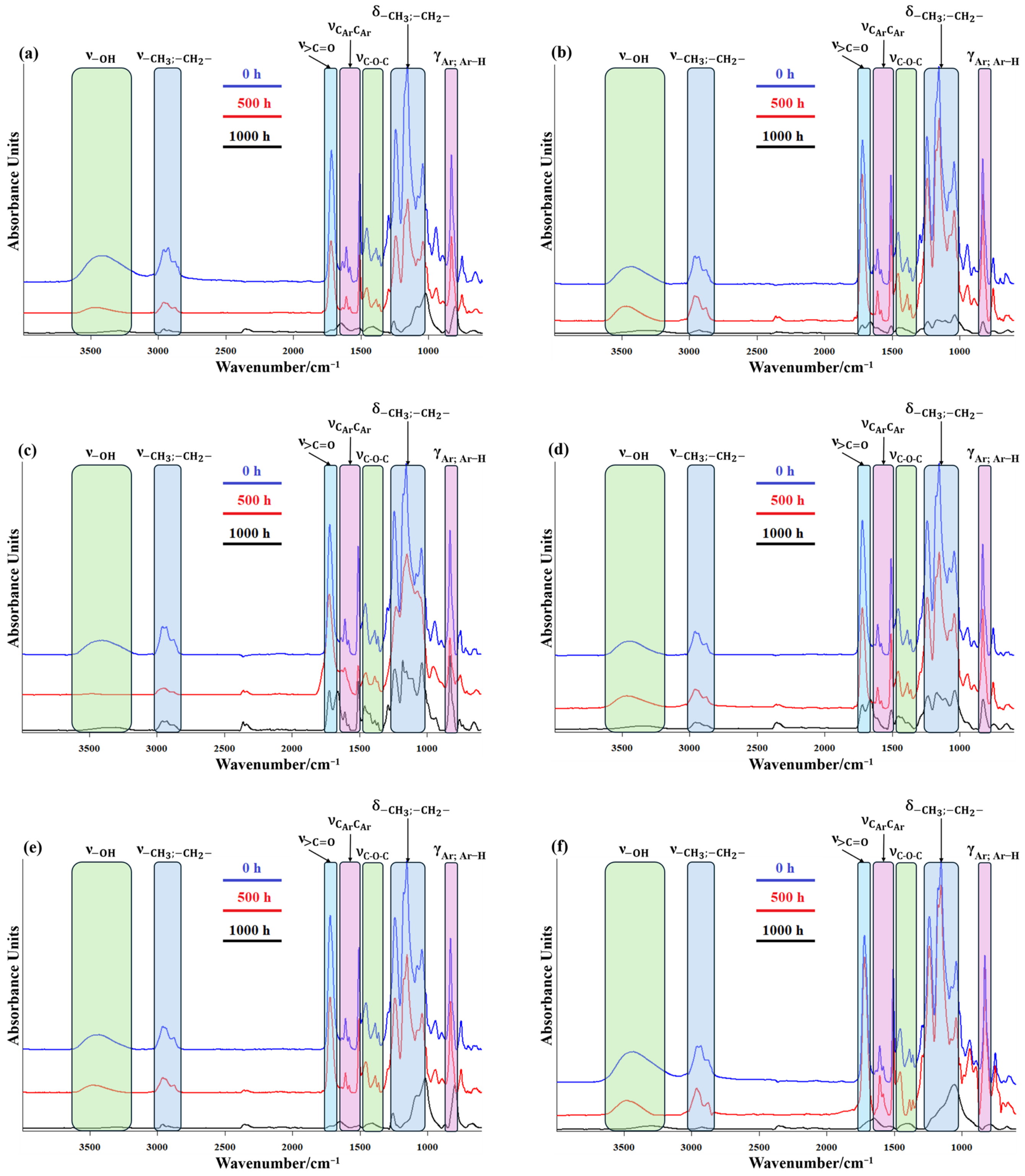

Figure S1. FTIR spectra of composite materials with HEMA as an active diluent recorded before ageing and after 500 and 1000 h of accelerated ageing: (a) unmodified composite (control), (b) composite containing ZnO, (c) composite containing benzethonium chloride (BEN), (d) composite containing $CuSO_4$, (e) composite containing nanosilver (Ag), and (f) composite containing zinc methacrylate (metZnO).

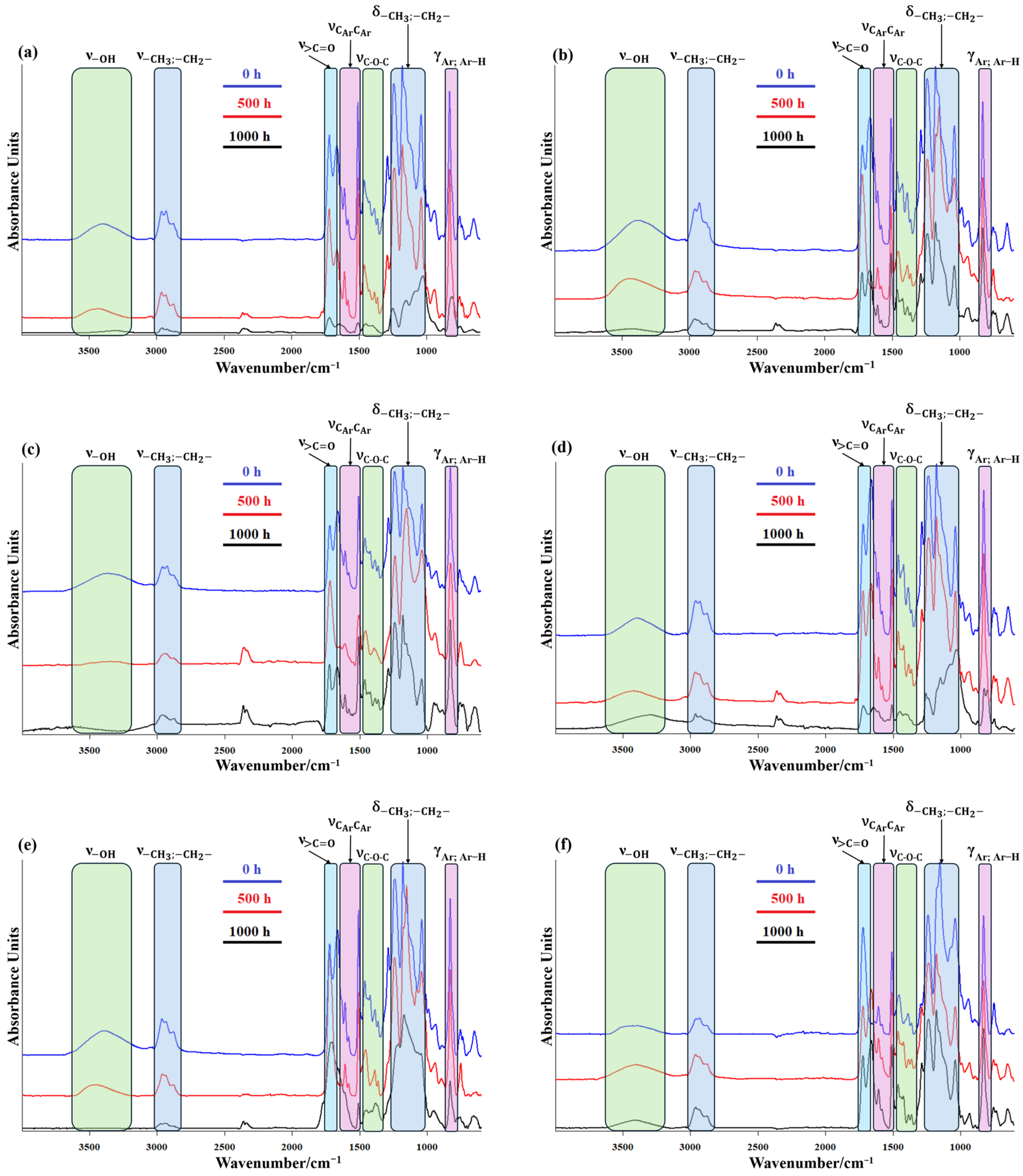

Figure S2. FTIR spectra of composite materials with NVP as an active diluent recorded before ageing and after 500 and 1000 h of accelerated ageing: (a) unmodified composite (control), (b) composite containing ZnO, (c) composite containing benzethonium chloride (BEN), (d) composite containing $CuSO_4$, (e) composite containing nanosilver (Ag), and (f) composite containing zinc methacrylate (metZnO).

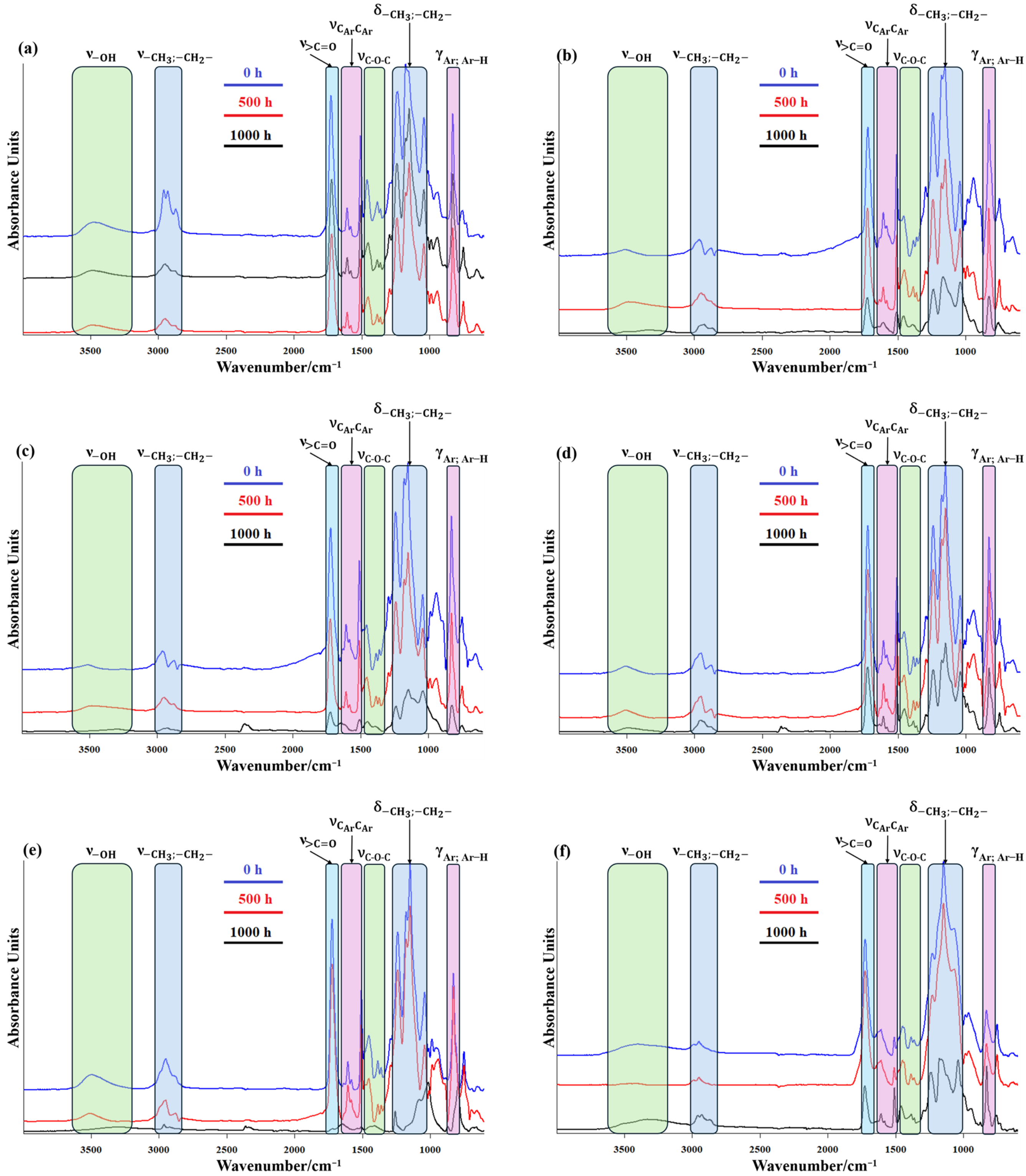

Figure S3. FTIR spectra of composite materials with MMA as an active diluent recorded before ageing and after 500 and 1000 h of accelerated ageing: (a) unmodified composite (control), (b) composite containing ZnO, (c) composite containing benzethonium chloride (BEN), (d) composite containing $CuSO_4$, (e) composite containing nanosilver (Ag), and (f) composite containing zinc methacrylate (metZnO).

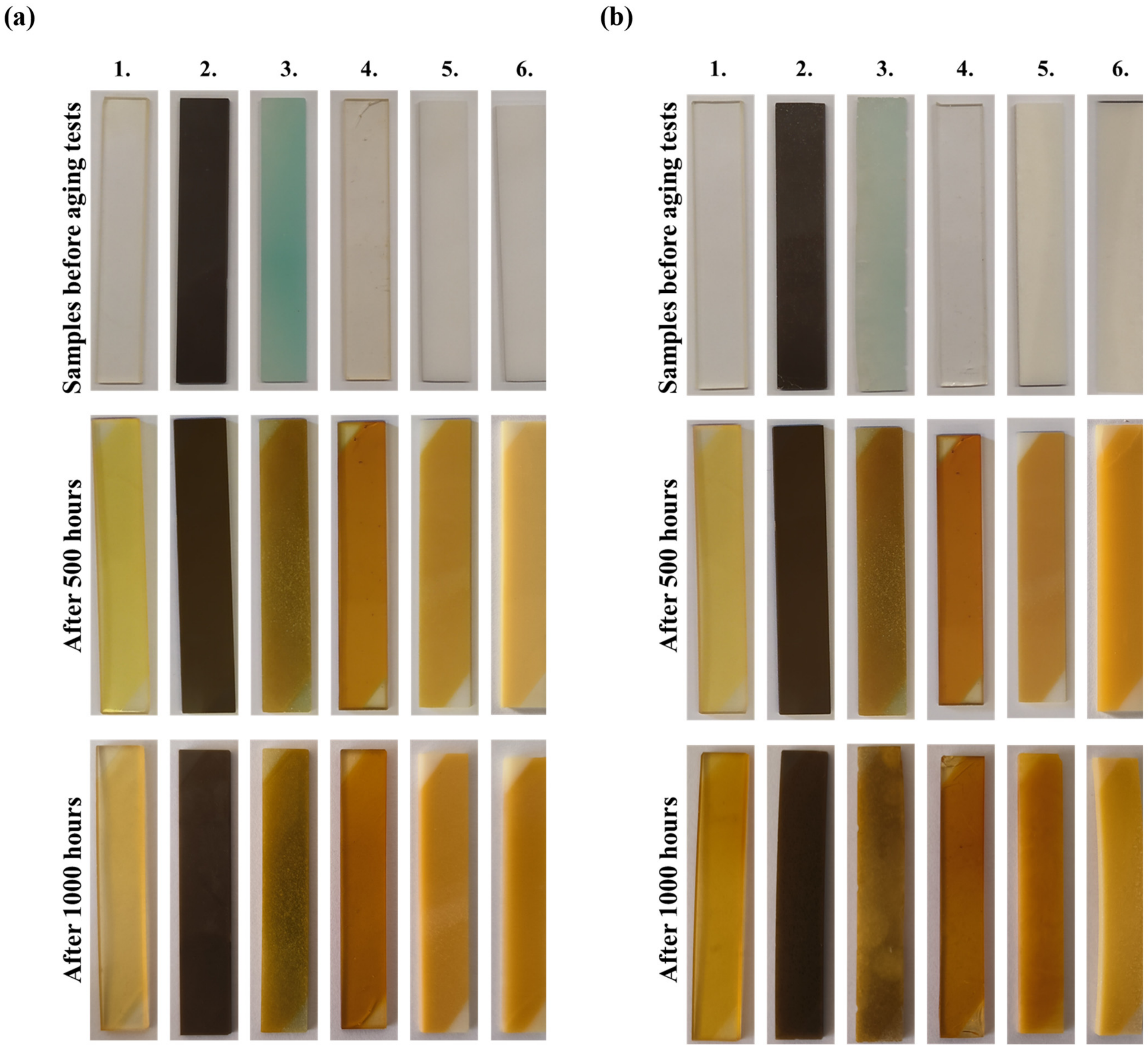

Figure S4. Color changes of AEH- and MMA-based composite materials before ageing and after 500 and 1000 h of accelerated ageing. (a) AEH-based composites; (b) MMA-based composites. Sample designations: (1) unmodified composite (control), (2) Ag, (3) $CuSO_4$, (4) BEN, (5) ZnO, and (6) metZnO.

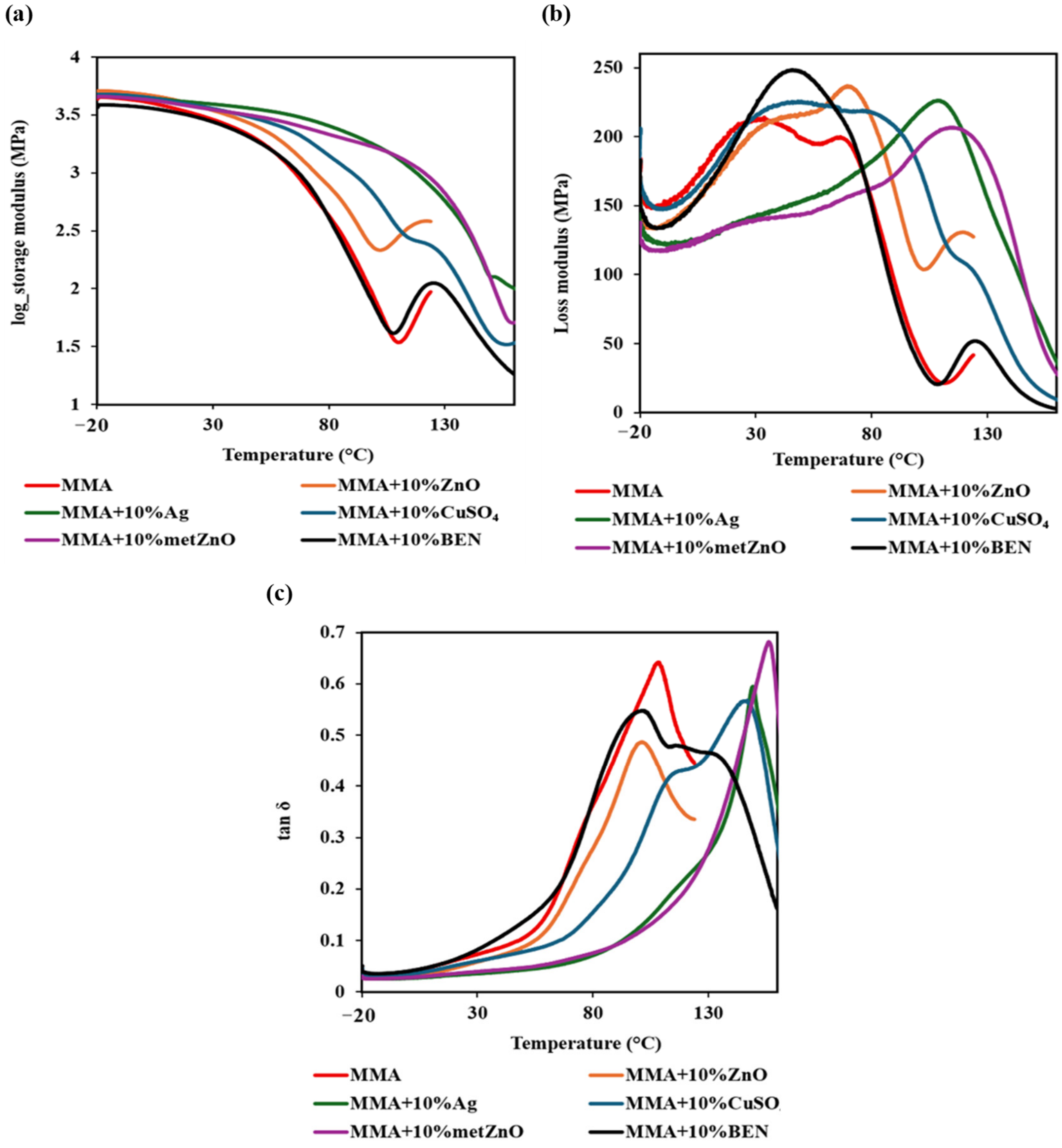

Figure S5. Dynamic mechanical analysis (DMA) curves of MMA-based composite materials: (a) storage modulus (E′), (b) loss modulus (E″), and (c) tangent delta (tan δ).

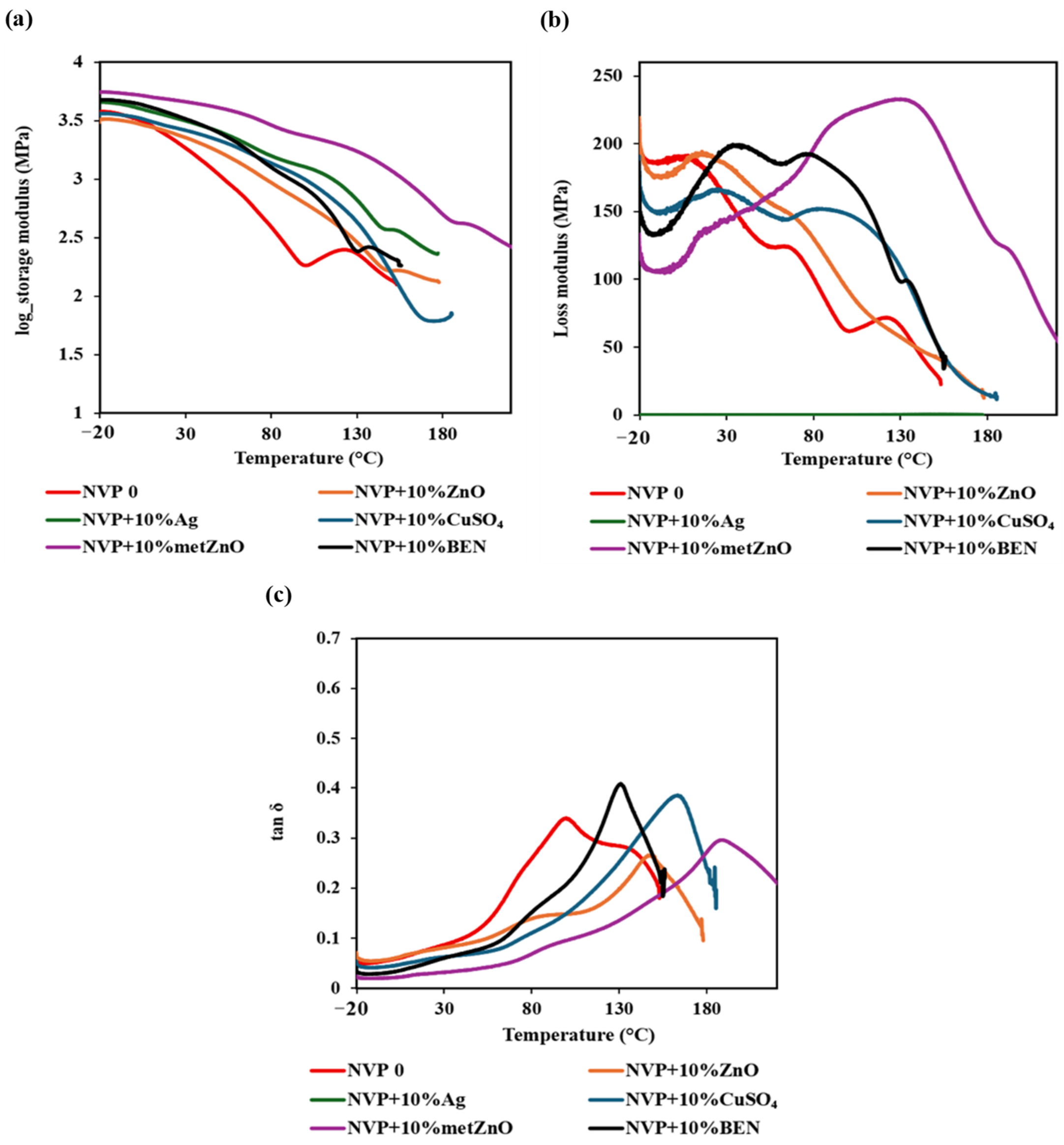

Figure S6. Dynamic mechanical analysis (DMA) curves of NVP-based composite materials: (a) storage modulus (E′), (b) loss modulus (E″), and (c) tangent delta (tan δ).

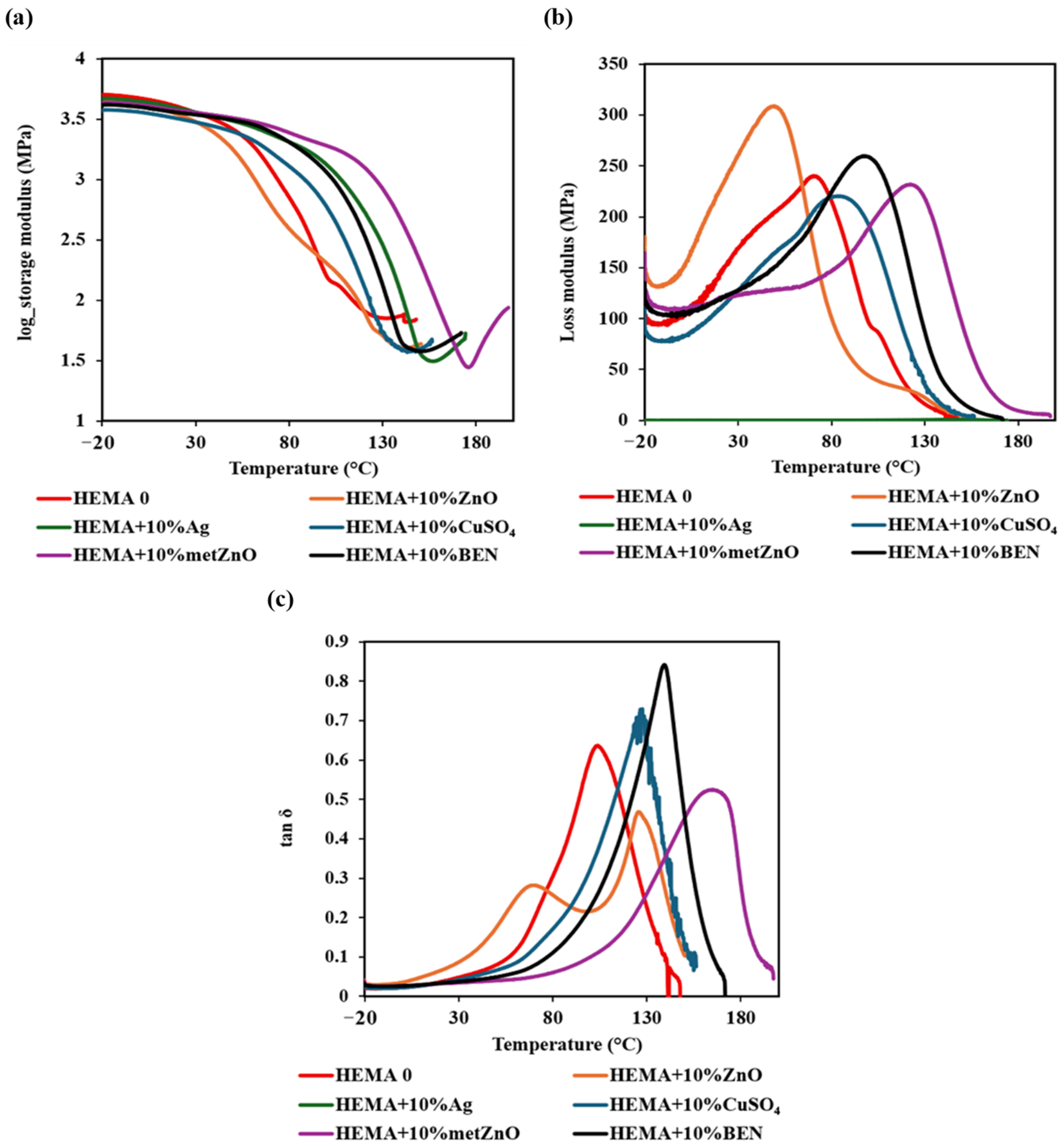

Figure S7. Dynamic mechanical analysis (DMA) curves of HEMA-based composite materials: (a) storage modulus (E′), (b) loss modulus (E″), and (c) tangent delta (tan δ).
